# Assessing local service providers’ needs for scaling up MHPSS interventions for Ukrainian refugees: Insights from Poland, Slovakia, and Romania

**DOI:** 10.1017/gmh.2024.113

**Published:** 2024-12-06

**Authors:** Marianna Purgato, Monica Bartucz, Giulia Turrini, Beatrice Compri, Eleonora Prina, Federica Patania, Emrah Kucukozkan, Anna Zubachova, Martin Mňahončak, Katarína Čavojská, Olena Koval, Gabriela Lupea, Vitalii Klymchuk, Natalie Maximets, Roberto Mediavilla, José Luis Ayuso-Mateos, Marit Sijbrandij, Els van der Ven, Iryna Frankova, Corrado Barbui

**Affiliations:** 1 World Health Organization Collaborating Centre for Research and Training in Mental Health and Service Evaluation, Department of Neurosciences, Biomedicine and Movement Sciences, Section of Psychiatry, University of Verona, Verona, Italy; 2 Department of Clinical Psychology and Psychotherapy, Babeș-Bolyai University, Cluj-Napoca, Romania; 3 International Medical Corps, Warszawa, Poland; 4 TENENET o.z., Senec, Slovakia; 5 Asociatia PHONEO, Cluj Napoca, Romania; 6 Department of Social Science, University of Luxembourg, Esch-Belval Esch-sur-Alzette, Luxembourg; 7 Department of Clinical, Neuro- and Developmental Psychology, Amsterdam Public Health Institute and World Health Organization Collaborating Center for Research and Dissemination of Psychological Interventions, Vrije Universiteit Amsterdam, Amsterdam, The Netherlands; 8 Department of Psychiatry, Universidad Autónoma de Madrid, Madrid, Spain; 9 Centro de Investigación Biomédica en Red de Salud Mental (CIBERSAM), Instituto de Salud Carlos III, Madrid, Spain; 10 Instituto de Investigación Sanitaria – Hospital Universitario La Princesa, Madrid, Spain; 11 ARQ Centrum 45, Oegstgeest, The Netherlands

**Keywords:** mental health, refugee, self-help, trauma

## Abstract

Providing Mental Health and Psychosocial Support interventions (MHPSS) for forcibly displaced Ukrainians in Central and Eastern Europe poses numerous challenges due to various socio-cultural and infrastructural factors. This qualitative study explored implementation barriers reported by service providers of in-person and digital MHPSS for Ukrainian refugees displaced to Poland, Romania and Slovakia due to the war. In addition, the study aimed to generate recommendations to overcome these barriers. Semi-structured Free List and Key Informant interviews were conducted using the Design, Implementation, Monitoring and Evaluation protocol with 18 and 13 service providers, respectively. For in-person interventions, barriers included stigma, language, shortage of MHPSS providers, lack of financial aid and general lack of trust among refugees. For digital MHPSS, barriers included generational obstacles, lack of therapeutic relationships, trust issues, and lack of awareness. Recommendations included advancing public health strategies, organizational interventions, building technical literacy and support, enhancing the credibility of digital interventions and incorporating MHPSS into usual practice. By implementing the recommendations proposed in this study, policymakers, organizations and service providers can work towards enhancing the delivery of MHPSS and addressing the mental health needs of Ukrainian refugees in host countries, such as Poland, Romania and Slovakia.

## Graphical abstract

**Figure:**
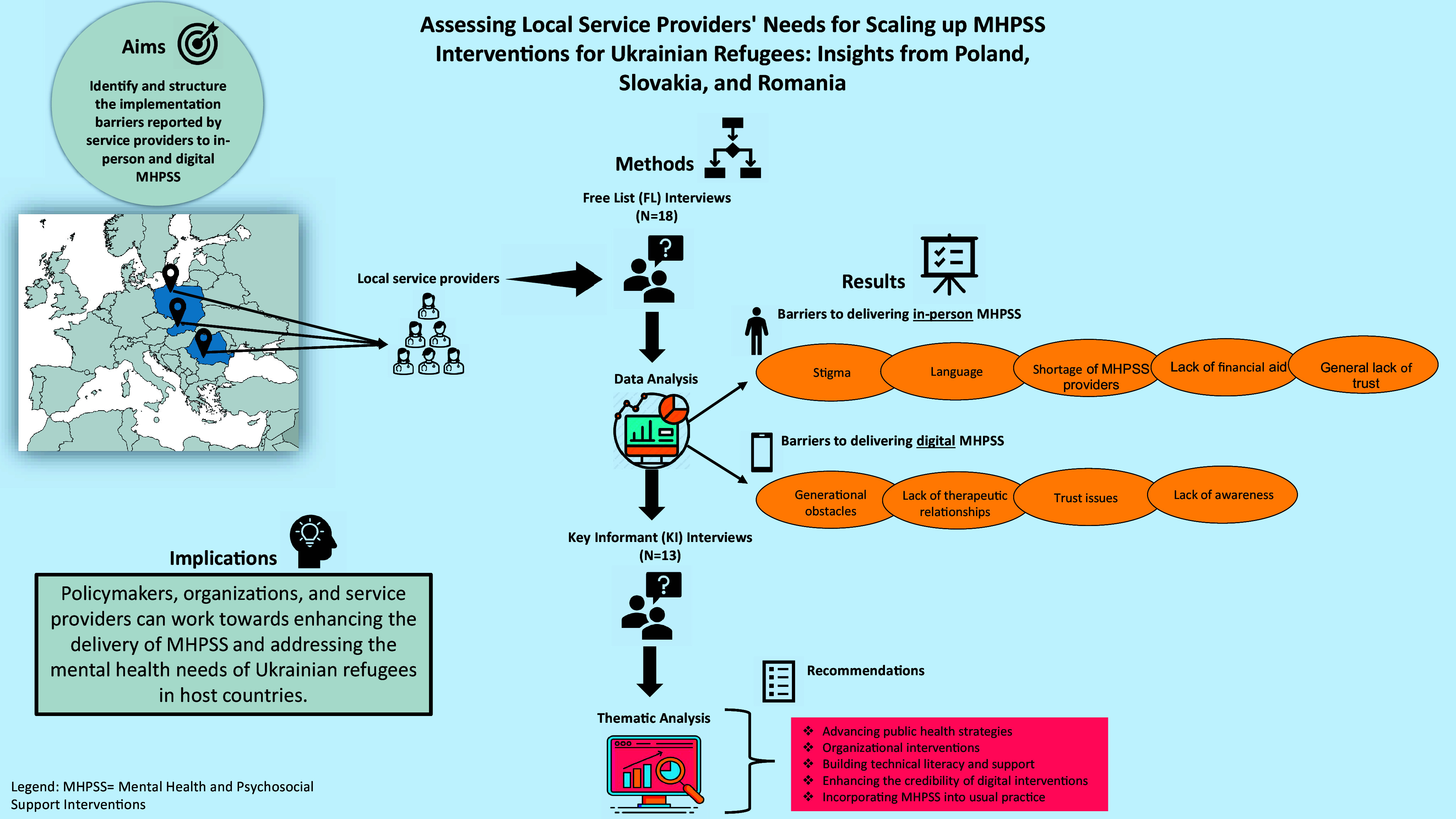

## Impact Statements

Since the Russian invasion of Ukraine, millions of Ukrainians have been forced to flee their homes, with many seeking refuge in neighboring countries such as Poland, Romania and Slovakia. Host nations’ healthcare systems are struggling to meet the psychological needs of millions of displaced individuals. This qualitative study is the first to explore the barriers and the recommendations to overcome them in the implementation of in-person and digital Mental Health and Psychosocial Support interventions (MHPSS) from the unique perspective of local service providers. Results indicate barriers, such as stigma and language barriers, systemic challenges, lack of MHPSS professionals, financial barriers and lack of trust. Among the strategies to overcome these barriers, service providers mentioned strengthening collaborative and coordinated MHPSS responses, as well as training initiatives for specialists, helpers and lay workers. Our results have significant implications to guide healthcare providers, policymakers and relevant authorities in addressing the mental health needs of displaced individuals and in planning implementation strategies for MHPSS.

## Introduction

The full-scale invasion of Ukraine by Russia has precipitated one of the largest humanitarian crisis in Europe since World War II, with millions of Ukrainians forced to flee their homes in search of safety and refuge. The escalating conflict has continuously increased displaced people, with the majority seeking shelter in neighboring countries such as Poland, Slovakia, Romania, Hungary, Moldova and Belarus, but also countries further away such as Germany, Italy and the Netherlands (IOM, 2024; UNHCR, 2024).

Refugees – including forcibly displaced Ukrainian populations – can be exposed to traumatic events associated with armed conflicts, such as bombardment, destruction of homes or war crimes. This raises the probability that they develop mental health disorders (Turrini et al., 2017). The World Health Organization (WHO) has determined that the prevalence of common mental disorders such as depression, anxiety and post-traumatic stress disorder (PTSD) in war regions is around 22%, that is, being conservative, five times higher than the prevalence in the general population (Charlson et al., 2019). In addition to challenging mental health, humanitarian crises place significant strains on the healthcare systems of the host nations and the mental health services available in these countries can only partially fulfill the psychological requirements of the millions of displaced people (WHO, 2013; Jordan et al., 2021; Troup et al., 2021; Papola et al., 2024). Moreover, mental health systems were not prepared to respond to the psychological consequences of the war with appropriate implementation models and crisis plans (Goto et al., 2023; Seleznova et al., 2023).

In response to this gap, the U-RISE project (Ukraine’s displaced people in the EU: Reach out, Implement, Scale-up and Evaluate interventions promoting mental wellbeing) aims to improve the mental health outcomes of Ukrainian refugees by implementing evidence-based psychosocial interventions developed by the WHO for populations affected by adversities. These interventions include Self-Help Plus (SH+) (Epping-Jordan et al., 2016) and its digital version *Doing What Matters in Times of Stress* (DMW), Problem Management Plus (PM+) (Dawson et al., 2015) and Psychological First Aid (PFA) Chatbot (WHO et al., 2011; Frankova et al., 2022). All these interventions were found effective in preventing and/or reducing symptoms of common mental health problems across vulnerable population groups (Koesters et al., 2018; Turrini et al., 2021; Schäfer et al., 2023; Cadorin et al., 2024).

Despite the clear need and the availability of WHO-developed interventions, successful implementation of Mental Health and Psychosocial Support interventions (MHPSS) for refugees faces significant barriers (Dickson et al., 2024). Previous studies have identified systemic barriers to implementing and scaling up MHPSS for refugees worldwide (Lotito et al., 2023; Dickson et al., 2024). The most frequent barriers are limited access to mental health services, insufficient funding, stigma and discrimination, language barriers, cultural differences and lack of culturally appropriate interventions, lack of trained and skilled mental health professionals and limited coordination among stakeholders involved in MHPSS efforts (Echeverri et al., 2018; Troup et al., 2021; Dickson et al., 2024). A comprehensive approach is needed to address these barriers and effectively implement MHPSS for refugees (Troup et al., 2021). Recent studies have focused on identifying the mental health needs among Ukrainian refugees primarily from the perspective of the refugees themselves. These studies showed that Ukrainian refugees, particularly those affected by the Russian-Ukrainian war, face significant mental health challenges (Rizzi et al., 2022; Asanov et al., 2023; Buchcik et al., 2023; Chudzicka-Czupała et al., 2023; Vitruk, 2023), and that large-scale implementation of MHPSS is urgently needed (Javanbakht, 2022). However, there is a notable gap in understanding what the specific challenges are in meeting the mental health needs of Ukrainian refugees from the perspective of service providers. One notable study conducted in the Czech Republic examined the challenges faced in implementing MHPSS for Ukrainian refugees from the perspective of mental health actors, including governmental, UN and national entities. The findings underscored several key barriers to implementing MHPSS for Ukrainian refugees in Czech Republic, such as an excessive demand placed on the National Healthcare System, low levels of mental health awareness among refugees, suboptimal monitoring and reporting practices concerning refugees’ mental health, concerns related to service provider burnout and suboptimal integration of international MHPSS guidelines into national emergency response plans (Budosan et al., 2023). More research is needed to understand the obstacles that impede both in-person and digital MHPSS for Ukrainian refugees across Europe. Furthermore, applying an implementation framework in this analysis is crucial, as it may offer a structured method for comprehending barriers at various levels and identifying specific factors hindering the effective scaling up of MHPSS for Ukrainian refugees.

Against this background, the present study had two aims. First, to identify and structure the implementation barriers reported by service providers to in-person and digital MHPSS using the socio-ecological and implementation models (Bronfenbrenner, 1979; Proctor et al., 2023). These models were adopted to contextualize individuals’ mental health within the complex range of social influences and environmental factors. Second, to gather recommendations on how to overcome these barriers.

## Methods

### Study design, participants and procedures

**Figure 1.**
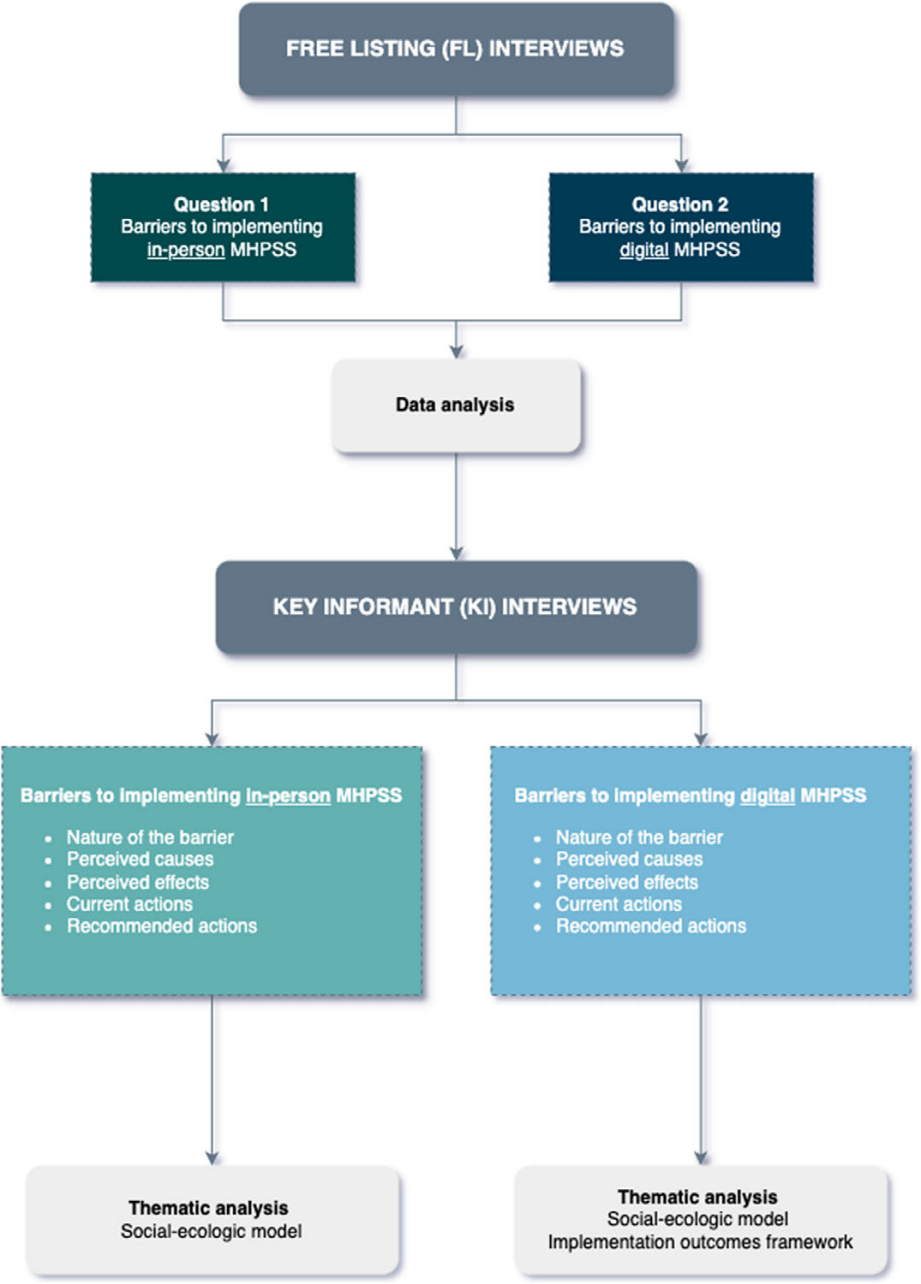
Study flow chart according to the DIME protocol.

We carried out a two-step qualitative study using an action research design (Speziale et al., 2011) following the DIME protocol (AMHR, 2013) (Figure 1). In the first step, we used semi-structured Free List (FL) interviews to prompt service providers, to identify obstacles encountered in delivering in-person and digital MHPSS for Ukrainian refugees displaced in Poland, Romania and Slovakia. In the second step, Key Informant (KI) interviews were held with service providers recommended by participants from the initial FL interviews due to their comprehensive understanding of the issues identified during the FL phase. The key informants described the nature, causes and consequences of these problems while also identifying ongoing efforts to address them and providing recommendations for overcoming these barriers.

This study included various adult service providers (≥18 years) proficient in English and working with the Ukrainian refugee community in Poland, Romania and Slovakia. When referring to service providers, we encompass a diverse group of professionals, such as psychologists, psychotherapists, social workers and lay workers. All of them had practical experience in delivering psychosocial support programs to Ukrainian refugees. They had daily experience in the field of migration, helping traumatized people to cope with demanding situations, such as social isolation, job loss, trauma exposure and psychological suffering. In addition, individuals associated with governmental or non-governmental organizations (NGOs) with a mission of mental health, social inclusion and education, and with experience coordinating psychosocial interventions for refugees were included in the study.

Sampling and recruitment strategies differed for FL and KI interviews. For the FL interviews, we used a maximum variation sampling method, balancing participants by gender and host country to address the diverse needs of service providers in various resettlement contexts (Drescher et al., 2021). Participants were recruited through NGOs affiliated with the U-RISE consortium: International Medical Corps Poland, Phoneo and Tenenet, located in Poland, Romania and Slovakia, respectively. Key informants within service provider communities were identified using a snowball sampling method (Naderifar et al., 2017). The sample size was determined based on the Applied Mental Health Research Group (2013) and WHO and UNHCR (2012) recommendations, involving 10–15 participants for both FL and KI interviews.

The study was conducted in accordance with the Declaration of Helsinki and received approval from the Institutional Review Board at the University of Verona (Study ID: 12a/2023). The study protocol was pre-registered in the Open Science Framework (OSF; https://osf.io/tmsk7).

Before the interview sessions, participants received an email outlining the study’s details and were requested to electronically sign both an informed consent for participation and a consent form for processing personal data. The interviews, conducted via Zoom with participants from Poland and Romania, lasted 40–50 min each. In Slovakia, the interviews were of similar duration and held in person.

### Free list interviews

The initial phase involved conducting individual FL interviews. Service providers were presented with a primary question: “What are the obstacles that make it hard to provide face-to-face psychological interventions to Ukrainian refugees living in [Poland/Romania/Slovakia]?” and a secondary question, namely: “What are the obstacles that make it hard to implement digital psychological interventions for Ukrainian refugees living in [Poland/Romania/Slovakia]?” The identified barriers were listed and participants were asked to describe each one and name a key informant who could provide more detailed information. The FL interview participants agreed to share the contact information of the KI if that person consented.

### Key informant interviews

The KI was tasked with providing additional information about the problems identified during the FL interviews. The interviewer inquired about various aspects of the selected problem, including its nature, perceived causes, effects on individuals and those close to them, current actions taken regarding it and potential courses of action if resources were available. The KI interviews were conducted using Zoom and transcribed for reference.

## Procedure

### Free list interviews

The interviews were conducted as follows. First, the interviewer asked participants to list as many problems as they could think of and provide a short description of each problem they identified, according to the DIME protocol. Participants were then repeatedly probed to list as many responses as possible until data saturation, where they indicated they could think of no more, or they thought that new data would have repeated what was already mentioned (thematic saturation) (Guest et al., 2006; Kerr et al., 2010). At the end of the interview, the interviewer asked the participants to think of someone knowledgeable of the problems they mentioned for the KI interviews.

In the analysis, participants’ responses were organized into a coherent list of barriers for each question, including the total number of individual interviews reporting each barrier. The interviewers collated all interviews and consolidated all data into a single list of responses for each FL question, including the number of different participants reporting each response (code frequency counts to reach saturation) (Morse, 1995; Guest et al., 2006; Hennink et al., 2017). The interviewers and the researchers conducted this process. The interviewers listed all the different responses from the interview forms, placing the participant ID number next to the response. If more than one person reported the same problem, all the relevant ID numbers were listed next to the response. If two or more participants referred to the same concept with different wording, the research team selected and recorded the wording they judged as most accurate and most likely to be understood by a member of the target population.

This list was subsequently reorganized based on the response frequency mentioned above. Based on this consolidated list, the research team selected the barriers that were discussed during the KI interviews. We decided to delve deeper into specific implementation obstacles based on two main criteria. First, we focused on barriers that were mentioned across all three countries. Second, we identified challenges mentioned by at least 50% of participants in at least one country.

### Key informant interviews

The KI interviewers coded the responses and created a summary sheet with subheadings for the investigated domains (e.g., nature of the problem and perceived causes). Researchers coded the frequency of each response category. The thematic analysis was performed using the NVivo software, following the step-by-step guide of Braun and Clarke (2006). For each identified in-person and digital implementation barrier, we coded the causes, consequences and recommendations according to the socio-ecological model of Bronfenbrenner (1979). Furthermore, the analysis of implementation barriers for digital interventions utilized the implementation outcomes framework (Proctor et al., 2011).

The choice of these specific theoretical frameworks is based on the following rationale. The socio-ecological framework of Brofenbrenner is particularly important for understanding the experiences of refugees, because it provides a comprehensive framework for examining the multiple layers of environmental influences that impact their lives, from the microsystem to the chronosystem. This holistic perspective is essential for refugee integration programs, as it underscores the need for multi-level interventions that address not just individual needs but also the broader social, economic and political contexts in which refugees are resettled (IASC, 2007). The implementation model of Proctor represents a natural subsequent step because it offers a structured approach to ensuring that MHPSS can be effectively delivered and sustained. In the data analysis, we linked the collected responses to the key components of these methodological frameworks.

### Data analysis

The data collection and analysis process employed various credibility strategies to ensure methodological integrity. These strategies included the following: methodological and investigator triangulation (FL and KI interviews, and participation in the study of a group of researchers with different backgrounds); documenting decision-making processes in an audit trail, in which all the recorded research phases and activities were verified and discussed in small-group meetings with experienced researchers (RMT, GT, MP); and supervising the data analysis process with guidance from experienced researchers (MP, CB, GT, RMT). For data analysis, we used a combined consensus and split coding. Researchers worked independently in coding the transcripts (MBB, GT, BC), with regular weekly meetings with expert researchers (MP, CB) to cross-check the coding schemes and review and discuss any questions. Transcripts were compared on a one-to-one basis for the first round, to ensure full alignment and reach a consensus. Then, we split coding keeping the regular weekly meetings for discussion.

For the thematic analysis, we used a hybrid deductive/inductive thematic analysis approach (Naeem et al., 2023). We used an open and inductive strategy for theme and recommendation generation, which were directly elaborated by study participants. At the same time, a theory-informed deductive application of themes to two existing frameworks was applied. The frameworks were identified in the literature as relevant for MHPSS implementation to vulnerable population groups (i.e., the socio-ecological model of Brofenbrenner and the implementation model of Proctor) (Bronfenbrenner, 1979; Proctor et al., 2023) and thoroughly discussed within the research team. This analytical approach aligns with those of other qualitative studies in this field (Morse, 1995; Hennink et al., 2017; Mediavilla et al., 2022).

## Results

### Sample characteristics

Sample characteristics are reported in Table 1, while demographic details for each participant are provided in Supplementary Table S1. A total of 18 participants were included in the FL interviews, with a mean age of 36.78 years (SD = 10.36) and 61% female participants. The distribution of participants included six service providers assisting the Ukrainian refugee population in Poland, Romania and Slovakia. For the KI interviews, we recruited 15 participants with an average age of 37.69 years (SD = 8.67). Female participants accounted for 85% of this group.

**Table 1.** Sample characteristics

	Free list (FL)	Key informant (KI)
	Interviews (*n* = 18)	Interviews (*n* = 13)
**Age group, *n* (%)**		
18–35	8 (44%)	4 (31%)
36–50	9 (50%)	8 (62%)
>50	1 (6%)	1 (8%)
**Female participants, *n* (%)**	11 (61%)	11 (85%)
**Employed, *n* (%)**	17 (94%)	13 (100%)
**Nationality, *n* (%)**		
Ukrainian	12 (67%)	3 (23%)
Polish	1 (6%)	1 (8%)
Romanian	2 (11%)	2 (15%)
Slovak	–	3 (23%)
Other	3 (17%)	4 (31%)
**Country of residence, *n* (%)**		
Poland	6 (33%)	5 (39%)
Romania	6 (33%)	4 (31%)
Slovakia	6 (33%)	4 (31%)
**Refugee status, *n* (%)**	10 (56%)	2 (15%)
**Area of expertise, *n* (%)**		
Healthcare and nursing	2 (11%)	–
Language and communication	2 (11%)	1 (8%)
Mental health and psychosocial support	7 (39%)	7 (54%)
Social services and support	7 (39%)	5 (39%)

### Free listing interviews

Barriers to implementing in-person and digital MHPSS, and the number and percentage of interviewees mentioning them in each country can be found in Table 2. The most commonly reported barriers were stigma related to mental health problems and the use of mental health services, language barriers, limited number of service providers, insufficient funding and lack of trust in mental health practitioners. Based on the interviews with FL, we identified 10 major barriers to implementing digital MHPSS across the target countries. The primary obstacles delineated in Table 2 exhibited consistent frequency across the three countries and included generational challenges, lack of therapeutic relationships, lack of trust and a notable lack of awareness regarding the existence and usability of such interventions. The identified barriers were linked to the socio-ecological framework of Bronfenbrenner (Table S1 in the Appendix), and the theoretical implementation framework of Proctor (Table 2S in the Appendix).

**Table 2.** Frequency of implementation barriers reported during the FL interviews

Implementation barriers	Total	Poland	Romania	Slovakia
	*n* (%)	*n* (%)	*n* (%)	*n* (%)
**Barriers to implementing in-person psychosocial interventions**				
Stigma	14 (78%)	5 (83%)	4 (67%)	5 (83%)
Mental health illiteracy	10 (56%)	3 (50%)	2 (33%)	5 (83%)
Language barriers	9 (50%)	2 (33%)	6 (100%)	1 (17%)
Lack of MHPSS professionals and infrastructure	8 (44%)	4 (67%)	2 (33%)	2 (33%)
Lack of funding	8 (44%)	3 (50%)	4 (67%)	1 (17%)
Lack of trust	7 (39%)	2 (33%)	3 (50%)	2 (33%)
Cultural barriers	6 (33%)	2 (33%)	2 (33%)	2 (33%)
Avoidance	4 (22%)	–	1 (17%)	3 (50%)
Transitory nature of refugees	4 (22%)	1 (17%)	3 (50%)	–
Transportation	2 (11%)	–	2 (33%)	–
**Barriers to implementing digital psychosocial interventions**				
Generational obstacles	9 (50%)	3 (50%)	4 (67%)	2 (33%)
Lack of therapeutic relationship	7 (39%)	4 (67%)	3 (50%)	–
Lack of trust	7 (39%)	1 (17%)	1 (17%)	5 (83%)
Lack of awareness	5 (28%)	–	4 (67%)	1 (17%)
Lack of internet access	4 (22%)	–	3 (50%)	1 (17%)
Stigma	2 (11%)	1 (17%)	1 (17%)	–
Limited efficacy for severe psychopathology	2 (11%)	1 (17%)	1 (17%)	–
Lack of priority	2 (11%)	–	2 (33%)	–
Lack of engaging digital design	2 (11%)	–	2 (33%)	–

*Note*: *N* = 18; *n* Poland = 6; *n* Romania = 6; *n* Slovakia = 6.

## Key informant interviews

### Implementation barriers to in-person psychosocial interventions

#### Mental health-related stigma

Service providers highlighted three primary contributors to the stigma surrounding mental health. At the societal level, cultural norms and beliefs emerged as a significant factor, encompassing the notion that mental health problems signify personal weakness and should be privately addressed without open dialog [“*Instead of getting some support from their own community or discussing them with a psychologist, they keep these problems private.”* (KIPS3)].

Another identified source of stigma was mental health illiteracy, characterized by a lack of understanding regarding the nature of mental health issues, available interventions and the roles of mental health specialists *[“(…) this lack of education contributes to confusion about the distinctions between the medical aspect of psychiatry and the psychological services.”* (KIRS2)]. The third cause of stigma was the apprehension of refugees about discrimination based on a mental health diagnosis. Service providers reported fears from refugees related to legal consequences, such as deportation or child custody issues, difficulties securing employment and social isolation [“(…) *how others will think about them, others could isolate them, they could not find a job.*” (KIRS2)]. At the individual level, the repercussions of stigma included a lack of trust in MHPSS professionals and reluctance to seek help [“(…) *people avoid psychological consultation.*” (KISS3)], leading to the adoption of maladaptive coping mechanisms [“(…) *alcohol consumption has increased* (KIPS3)], prolonged untreated mental health issues and the exacerbation of medical problems [“*(…) unfortunately, these problems will grow increasingly larger because chronic stress over a prolonged period has quite harmful effects, including on the organism.*” (KIRS2)]. Additionally, participants highlighted challenges for refugees in adapting to the host country [“*It could be harder for them to work, carry out their activities, and maintain close interpersonal relationships.*” (KIRS2)]. At the interpersonal level, stigma manifested in impaired social functioning of refugees [“(…) *they have problems within their families, their relationships get worse*” (KIRS2)]. At the organizational level, the presence of stigma presents challenges in the accurate identification and resolution of mental health issues. This challenge arises from a limitation in transparent communication during assessments, where Ukrainian refugees tend to minimize the presence of mental health problems.

### Language barriers

Language barriers constitute a significant challenge for service providers implementing MHPSS programs for Ukrainian refugees residing in Poland, Romania and Slovakia. Language is the primary tool for individuals to express their emotions, thoughts and feelings [“*Psychologists need to listen to every word you say to understand you.*” (KIPS2)]. The linguistic variations among the four cultures might impede in-depth communication between individuals. English is not commonly spoken at the necessary proficiency level among Ukrainian refugees and many service providers in the host countries [“(…) *it’s not really typical to use English at work” (*KIPS2)]. The ability to overcome language barriers is hindered by multiple causes, including a shortage of interpreters [(…) “*a key issue is the absence of interpreters when engaging with professionals.”* (KIPS3)], financial constraints and the high costs of interpretation services [“*One of our biggest challenges (…) was the cost of translation.”* (KIPS2)], as well as the lack of professional recognition of displaced Ukrainian mental health specialists that could effectively deliver MHPSS services to other Ukrainian refugees in the host countries [“(…) *we have the problem of not being able for Ukrainian refugees to integrate as professionals in the service providing industry.*” (KIPS2)]. In addition, the hope and prospect of returning to Ukraine has been reported as a source of hesitancy in learning the local language among Ukrainian refugees [*“Some refugees are reluctant to learn the language because it takes time, takes resources, but they are waiting to go home”* (KIPS2)]. These causes contribute to several consequences, notably untreated mental health problems such as anxiety, depression and PTSD among Ukrainian refugees [“*One problem which can be easily resolved with a couple of sessions, it gets bigger and like more severe and much harder to treat*” (KIPS2]. Furthermore, language barriers result in the isolation of Ukrainian communities, fostering feelings of loneliness, pessimism and stress [“*They feel more lonely because we cannot reach them.*” (KIRS3)]. Service providers experience demoralization, feeling useless and lacking motivation, leading to an inability to effectively provide MHPSS and engage the community of Ukrainian refugees [“*I felt so empty in a way, and so useless.*”(KIRS3)]. The burden falls disproportionately on a few Ukrainian-speaking mental health professionals, social workers and interpreters, exacerbating the shortage of MHPSS specialists. This is evident in long waiting times and a scarcity of Ukrainian-speaking personnel (“*The waiting queue is very long and (…) Ukrainian refugees get tired.*” (KIPS3)].

### Lack of MHPSS professionals and infrastructure

The lack of MHPSS professionals emerged as a critical impediment to service providers effectively implementing MHPSS. This barrier may be caused by the novelty of these services, suboptimal prioritization and insufficient funding [“*There is simply a poorly funded non-profit sector.*” (KISS4)]. Further challenges are language and cultural barriers, diverse career pathways for MHPSS professionals and restrictions on professional practice in host countries [“*Ukrainian psychologists are not allowed to work officially without a certification of the diploma.*” (KIPS1)]. Professional difficulties and constraints, such as the difficulty of certification, challenges in the profession itself, and limited training and education in MHPSS during crises, represent additional challenges. The consequences encompass reduced training capacities [“*We do not yet have the capacity to train peer advisors of other organizations from our resources.*” (KISS4)], leading to challenges in integrating foreign professionals into the system, lower service quality and accessibility with long waiting hours and frequent changes in mental health specialists [“*There are queues for 6 months of waiting for consultation.*” (KIPS5)]. [“*So far we haven’t been successful with the fact that we can educate and train these peer advisors and other organizations on a larger scale.*” (KISS4)]. This limits the ability to reach a large number of beneficiaries and places an undue burden on the few available Ukrainian-speaking mental health practitioners. For Ukrainian refugees, the consequences include enduring long-term mental health problems such as anxiety, depression and PTSD [“*Significantly deteriorating mental health not only in adults, but also in children.*” (KISS2)]. Other problems include intergenerational suffering, professional adaptation challenges and impairment in social functioning (e.g., family conflicts).

### Financial issues

Financial barriers include lack of adequate allocation of resources for the mental health sector with limited and short-time donor support [“*A lot of donors have cut down their funding.*” (KIPS3)] and high costs of private psychosocial care [“*In the private sector the fees are very high. While they receive some financial support, not everyone has a well-paying job to cover their needs and also their healthcare costs.*” (KIPS3)] (Figure 1S in the Appendix). The causes of these financial barriers are rooted in the state-level perception of MHPSS, marked by suboptimal prioritization, limited integration of mental health into the healthcare system and reduced state-level funding. In addition, funding challenges are linked to a general lack of knowledge on accessing available funds. The consequences are wide-reaching, burdening NGOs and small organizations that compete for limited funding, resulting in sustainability challenges [“*The financial challenges impact service providers significantly. Much of our energy, which could be directed towards program development and improving quality, is instead consumed by the stressful process of budgeting.*” (KIPS5)]. Service providers within these organizations reported low remuneration [“*The payment is very low.*” (KIRS3)], with a potential impact on service quality [“*It reflects on quality, unfortunately.*” (KIRS3)]. The burden extends to the few available Ukrainian-speaking mental health practitioners. The shortage of MHPSS specialists is reported to be associated with long waiting hours and frequent changes in mental health specialists [“*There are queues for 6 months of waiting for consultation.*” (KIPS5)].

### Lack of trust in mental health support/practitioners

The lack of trust emerges as a barrier for service providers (Figure 1S in the Appendix). Cross-cultural challenges, language barriers and a gap in legal knowledge may further complicate establishing trust. War-related trauma and perceived vulnerability may have heightened alertness to danger and a perceived susceptibility to exploitation among affected refugees. [“*(…) a fear that if I say something they will send me back to Ukraine.*” (KISS1)], or having children taken away [“*Ukrainian families were hesitant to seek help, as they were under the impression that Polish specialists might take their children away.*” (KIPS 4)]. Additionally, rapid changes in administrative procedures and suboptimal experiences with MHPSS may contribute to the lack of trust. The consequences of the lack of trust are profound, leading to adaptation challenges in making relevant life decisions, social functioning impairment and communication barriers generating hesitancy to disclose problems. There is also hesitancy to accept a diagnosis, resulting in difficulties in taking steps towards treatment [“*They didn’t want to accept that their child has autism or any kind of disorder*” (KIRS1)]. MHPSS workers are often needed to offer prolonged psychosocial support with initial sessions focused on building trust before addressing the core mental health concerns [“*This is why, for example, we don’t just have one consultation, but rather 8 or 10 consultations.*” (KIRS1)].

### Strategies to overcome the barriers

Service providers reported a comprehensive set of recommendations to address barriers to implementing in-person MHPSS for Ukrainian refugees in Poland, Romania and Slovakia (Table 1S in the Appendix outlines). Recommendations are categorized according to the socio-ecological model of Bronfenbrenner, indicating the level at which each recommendation could be implemented. These levels encompass public policy, organizational, and individual provider perspectives (Table 4S in the Appendix). For example, service providers mentioned enhancing funding opportunities and accessibility, incorporating Ukrainian specialists in organizations and projects providing MHPSS and language education initiatives for Ukrainian refugees.

### Implementation barriers to digital psychosocial interventions*:* Problem, causes and consequences

Service providers highlighted the following four key challenges associated with implementing the MHPSS in digital mode: (1) generational obstacles, (2) lack of therapeutic relationship, (3) lack of trust and (4) lack of awareness of the existence of MHPSS (see Table 2S and Figure 2S in the Appendix). Among these issues, there seems to be a common thread of problems related to acceptability and the evaluation of intervention appropriateness, both from the perspective of service providers and, as hypothesized, by Ukrainian refugees and end-users. Older generations of Ukrainians may be challenged by technical illiteracy [“*People who are from Ukraine and who are, for example, 50+ don’t know how to use mobile applications, or when we talk about a chatbot program*” (KISS1)] and lack of familiarity with new technologies. [“*It is possible that some of them only have ordinary phones with keyboards.*” (KISS1)]. This perception leads to a sense of poor therapeutic relationship, ineffective intervention and reduced self-efficacy in overcoming these challenges. The resultant effect is that it is difficult to reach and engage Ukrainian refugees with digital MHPSS. At the implementation level, service providers may encounter difficulties in adopting such interventions, rendering them unfeasible and challenging to integrate into existing services. At the clinical level, implementation barriers may lead to isolation and increased vulnerability, as well as untreated mental health problems. Regarding the lack of therapeutic relationship, the cultural perception of Service Providers (SPs) and Ukrainian Refugees (URs) regarding MHPSS and the social determinants (i.e., exposure to war-related trauma) may impact the perceived inadequacy of the intervention [“*People who have experienced trauma are more likely to benefit from physical contact, rather than talking with a chatbot.*” (KIPS1)]. These challenges may have consequences on the feasibility, effectiveness, penetration and sustainability of MHPSS. The lack of trust towards digital MHPSS is accompanied by similar consequences, including lack of uptake, development and promotion). According to SPs, the absence of direct contact with a professional significantly contributes to the lack of trust in digital interventions. Other factors mentioned by SPs are concerns for user safety and worries about data security [“*It’s quite stressful to think that someone might have access to details of your card and bank account*” (KIRS4)], the perception of low-quality design, the poor quality of translation into Ukrainian and the perception of limited scientific evidence and efficacy of digital interventions. This may result in difficulties in adopting, developing, integrating and maintaining these interventions within an existing service system.

Finally, the lack of awareness refers to SPs and URs lacking knowledge about evidence-based digital MHPSS. One of the major causes of this obstacle is the lack of promotion of digital interventions. Consequently, individuals who could benefit from digital interventions may instead turn to traditional mental health services, increasing the demand for specialists. Lack of awareness can lead to challenges in determining whether to adopt, integrate and sustain MHPSS within the system. From a clinical perspective, this may lead to consequences such as prolonged and aggravated mental health problems for URs, dysfunctional coping mechanisms and social impairment (Figure 2S in the Appendix).

### Strategies to overcome the barriers

Service providers described a comprehensive set of recommendations to overcome barriers to implementing digital MHPSS (Table 3S in the Appendix). These recommendations, organized based on the socio-ecological model, indicate the suitable level for implementation and cover policy, organizational and individual provider perspectives. For example, service providers mentioned creating a comprehensive national plan for the seamless integration of digital interventions into mental health services, as well as promoting awareness about the benefits and usage of digital tools.

## Discussion

This study aimed to identify barriers and strategies to overcome them reported by service providers in implementing in-person and digital MHPSS for Ukrainian forcibly displaced persons in Poland, Romania and Slovakia.

Service providers identified several barriers to implementing in-person MHPSS for Ukrainian refugees in Poland, Romania and Slovakia. The most frequent barriers included stigma, language, shortage of MHPSS providers and consequently high workload, lack of financial aid and general lack of trust among refugees. These results align with previous studies focused on access to mental care services for refugees and asylum seekers in various host countries (Javed et al., 2021; Javanbakht, 2022). For example, a study conducted with mental health professionals working with refugees in Jordan identified similar barriers. Through semi-structured and unstructured interviews, mental health professionals were asked about potential barriers according to a list developed from a scientific literature review (Al-Soleiti et al., 2021). In line with our results, stigma, financial limitations, shortage of mental health personnel and burnout were the barriers reported by participants (Al-Soleiti et al., 2021). Similar results were found in a qualitative study by Bawadi et al. (2022), which identified stigma, social discrimination and accessibility of mental health services as the main barriers to accessing MHPSS for Syrian refugees hosted in Jordan. Another important consideration relates to the potential differences in the prevalence of mental disorders and access to mental health services within the group of Ukrainian refugees. In this regard, epidemiological data on war-exposed adolescent Ukrainian refugees show increased levels of moderate to severe depression, anxiety and clinically relevant psychological trauma (Goto et al., 2024). This confirms a substantial burden in refugee populations of different age ranges and a consequent need for investment in mental health care. In Poland, Slovakia and Romania, healthcare services are provided free of charge to refugees, covering both primary and emergency care. Furthermore, the IOM has been actively engaged in these countries, implementing comprehensive programs to address the diverse health needs of Ukrainian refugees. These efforts by the IOM include not only medical assistance but also support in areas such as mental health, housing, (psycho)education and social integration. This ensures that Ukrainian refugees receive the care and resources necessary for their health across countries and successful adaptation in their host communities (IOM, 2024).

To overcome the barriers reported above, our participants formulated several recommendations. Key strategies include the development of state-level policies and national plans to organize MHPSS responses, enhancing funding opportunities for MHPSS provision, and accessibility through financial support and donor mobilization. Other strategies included incorporating specialists with a Ukrainian background into host country mental healthcare systems, fostering collaborative MHPSS responses between practitioners and stakeholders, facilitating group and community-based activities, training MHPSS specialists and helpers, promoting digital MHPSS and offering language education. Many of these recommendations are aligned with the contents of a Lancet Commission on Migration and Health (Abubakar et al., 2018) and with a WHO document focused on the mental health of refugees and migrants (WHO, 2023b). This WHO document reported barriers and facilitators to deliver MHPSS for professionals working with refugees and asylum seekers, and policy and clinical considerations organized according to the social determinants of mental health framework developed by Lund et al. (2018) and the socio-ecological theory of Bronfenbrenner (1979), Purgato et al. (2017). Practitioners reported difficulties related to the workload, language barriers, stigma towards mental health conditions, problems in retaining mental health staff and emotional exhaustion of staff. Recommendations highlighted the importance of training and clinical supervision, liaison and collaboration with local stakeholders, the attention paid to local contexts and cultural competency (WHO, 2023b). Next, service providers identified several barriers to implementing digital MHPSS for Ukrainian refugees. These included generational obstacles such as differing levels of comfort with technology among different age groups, the lack of a therapeutic relationship within digital interventions, which may hinder trust, perceived concerns related to the efficacy and trustworthiness of digital MHPSS, data security concerns and a general lack of awareness of the benefits of these interventions. In a narrative review by Torous et al. (2018) about the use of mobile phones in a clinical context, participants indicated privacy, confidentiality and anxiety about third-party use of personal information as perceived barriers to MHPSS access (Mabil-Atem et al., 2024). Furthermore, while digital interventions are becoming important and popular globally, there exists a significant risk that older people are left behind. Older people often face challenges in adapting to new technologies, so they may encounter barriers that limit their access to digital interventions. This is particularly relevant for the refugee population. This could exacerbate existing healthcare inequalities, leaving older refugee populations at a distinct disadvantage in receiving timely and effective psychosocial support. Ensuring inclusivity in the adoption of digital health solutions is crucial to prevent further marginalization of refugee older adults and to promote equitable healthcare access for all (Hollis et al., 2015; Seifert et al., 2019). The cost of devices was a factor that determined their decision-making about the uptake of digital health interventions. Additional barriers included exposure to racism, discrimination and stigmatization (Romao et al., 2021). Recommendations to address these barriers include developing national plans and gaining state-level support for integrating digital interventions into mental health systems, promoting technical literacy through educationand enhancing the credibility of digital interventions through efficacy research. They also advised disseminating and improving acceptability and quality through focus groups and MHPSS testing, providing training to MHPSS practitioners and raising awareness through various promotional strategies, as well as overcoming implementation challenges by integrating digital interventions into routine practice. These strategies have also been cited as a means for improving mental health literacy and social connectedness among refugees (Ekblad et al., 2024). For example, potential protective factors may be social support through communication (e.g., phone, WhatsApp, email and video calls) with significant persons who are separated. Another protective factor may be the increase in refugees’ perceived health and mental health literacy through the participatory approach (Leask et al., 2019). To work with the topic of healthy communication in the field demands knowledge and experience, and this is supported by the WHO statement that “addressing these determinants and enhancing the health of migrants, refugees and other displaced populations are essential goals for global health and sustainable development” (WHO, 2023a). In this regard, an important consideration stated in the WHO manual on the implementation of psychosocial interventions is that remote delivery of MHPSS should, in any case, not be seen as a replacement for in-person support (WHO, 2024). Relying on digital technologies only risks excluding poor or marginalized people without access to the internet or a private phone. A key objective in organizing and delivering interventions is to offer different delivery options (also in parallel) to support a broad range of needs and preferences (WHO, 2022; WHO, 2024).

Our study has several limitations. Although the number of participants in our study is consistent with the practical guidance reported in the literature (Guest et al., 2006; WHO and UNHCR, 2012; Keddem et al., 2021), and with other qualitative studies conducted in the field of global mental health (Atiq et al., 2024; Elnasseh et al., 2024), the overall number of study participants was limited. Therefore, our findings and recommendations should be interpreted with caution, as they cover different countries with potential differences in the implementation and delivery of mental health services. Additionally, we note an underrepresentation of male participants, especially in the key informant phase of the study. Future studies should strive for gender inclusivity to ensure a comprehensive understanding of the challenges and barriers experienced by both male and female participants. Furthermore, it is important to recognize that this study is qualitative, relying on interviews to gather data. While qualitative studies provide valuable insights into the lived experiences of individuals, they may be influenced by the researchers’ interpretations and biases. Another important limitation is that the questions addressed to the participants focused on the implementation barriers of MHPSS in general. With this approach, we intended to stay close to the DIME protocol, simplify the questions addressed and inform the implementation barriers for MHPSS in general. However, valuable insights could have been gained by focusing on the barriers to implementing specific in-person and digital intervention protocols.

In conclusion, this paper outlines a comprehensive set of barriers and recommendations to address these barriers, spanning multiple levels of intervention. From policy initiatives to organizational strategies and individual provider perspectives, there is a need for coordinated efforts to create an enabling environment for MHPSS delivery, particularly in situations of crisis. Promoting awareness, enhancing training programs, improving access to resources and fostering collaboration across sectors are key strategies to overcome implementation challenges. Through an in-depth understanding of the nature of the reported barriers and challenges in implementing evidence-based strategies, we can ensure that vulnerable populations receive the support they need to overcome adversity and rebuild their lives.

## Supporting information

Purgato et al. supplementary material Purgato et al. supplementary material

## Data Availability

The data that support the findings of this study are available upon motivated request to the corresponding author.

## Supplementary material

The supplementary material for this article can be found at http://doi.org/10.1017/gmh.2024.113.

## References

[r1] Abubakar I, Aldridge RW, Devakumar D, Orcutt M, Burns R, Barreto ML, Dhavan P, Fouad FM, Groce N, Guo Y, Hargreaves S, Knipper M, Miranda JJ, Madise N, Kumar B, Mosca D, McGovern T, Rubenstein L, Sammonds P, Sawyer SM, Sheikh K, Tollman S, Spiegel P, Zimmerman C and UCL–Lancet Commission on Migration and Health (2018) The UCL-Lancet Commission on migration and health: The health of a world on the move. Lancet 392 (10164), 2606–2654. 10.1016/S0140-6736(18)32114-7 30528486 PMC7612863

[r2] Al-Soleiti M, Abu Adi M, Nashwan A and Rafla-Yuan E (2021) Barriers and opportunities for refugee mental health services: Clinician recommendations from Jordan. Global Mental Health 8, e38. 10.1017/gmh.2021.36 34631114 PMC8482442

[r3] Applied Mental Health Research Group (AMHR) (2013) Design, Implementation, Monitoring and Evaluation of Cross-Cultural Trauma Related Mental Health and Psychosocial Assistance Programs: A User’s Manual for Researchers and Program Implementers. Baltimore: Johns Hopkins University Bloomberg School of Public Health. https://hopkinshumanitarianhealth.org/assets/documents/VOT_DIME_MODULE1_FINAL.PDF

[r4] Asanov Noha A-M, Asanov I and Buenstorf G (2023) Mental health and stress level of Ukrainians seeking psychological help online. Heliyon 9, e21933. 10.1016/j.heliyon.2023.e21933 38027618 PMC10658344

[r5] Atiq M, Nazir H, Rahman A, Malik A, Atif N and Surkan PJ (2024) Exploring preference for delivery methods for a psychosocial intervention for prenatal anxiety: A qualitative study from a tertiary care hospital in Pakistan. Global Mental Health (Cambridge, England) 11, e66. 10.1017/gmh.2024.59 38827335 PMC11140489

[r6] Bawadi H, Al-Hamdan Z, Khader Y and Aldalaykeh M (2022) Barriers to the use of mental health services by Syrian refugees in Jordan: A qualitative study. Eastern Mediterranean Health Journal = La Revue De Sante De La Mediterranee Orientale = Al-Majallah Al-Sihhiyah Li-Sharq Al-Mutawassit 28 (3), 197–203. 10.26719/emhj.22.030 35394051

[r7] Braun V and Clarke V (2006) Using thematic analysis in psychology. Qualitative Research in Psychology 3, 77–101. 10.1191/1478088706qp063oa

[r8] Bronfenbrenner U (1979) The Ecology of Human Development: Experiments by Nature and Design. Harvard University Press.

[r9] Buchcik J, Kovach V and Adedeji A (2023) Mental health outcomes and quality of life of Ukrainian refugees in Germany. Health and Quality of Life Outcomes 21 (1), 23. 10.1186/s12955-023-02101-5 36894946 PMC9996949

[r10] Budosan B, Castro J, Kortusova P and Svobodova I (2023) Challenges and opportunities for mental health and psychosocial support programming during Ukraine refugee crisis in Czechia. Intervention Journal of Mental Health and Psychosocial Support in Conflict Affected Areas 21 (2), 107. 10.4103/intv.intv_19_23

[r11] Cadorin C, Purgato M, Turrini G, Prina E, Cabral Ferreira M, Cristofalo D, Bartucz MB, Witteveen AB, Sijbrandij M, Papola D and Barbui C (2024) Mapping the evidence on psychosocial interventions for migrant populations: Descriptive analysis of a living database of randomized studies. Cambridge Prisms: Global Mental Health 11, e35. 10.1017/gmh.2024.33. 38572262 PMC10988138

[r12] Charlson F, van Ommeren M, Flaxman A, Cornett J, Whiteford H and Saxena S (2019) New WHO prevalence estimates of mental disorders in conflict settings: A systematic review and meta-analysis. The Lancet 394 (10194), 240–248. 10.1016/S0140-6736(19)30934-1 PMC6657025 31200992

[r13] Chudzicka-Czupała A, Hapon N, Chiang S-K, Żywiołek-Szeja M, Karamushka L, Lee CT, Grabowski D, Paliga M, Rosenblat JD, Ho R, McIntyre RS and Chen Y-L (2023) Depression, anxiety and post-traumatic stress during the 2022 Russo-Ukrainian war, a comparison between populations in Poland, Ukraine, and Taiwan. Scientific Reports 13 (1), 3602. 10.1038/s41598-023-28729-3 36869035 PMC9982762

[r14] Dawson KS, Bryant RA, Harper M, Kuowei Tay A, Rahman A, Schafer A and van Ommeren M (2015) Problem management plus (PM+): A WHO transdiagnostic psychological intervention for common mental health problems. World Psychiatry 14 (3), 354–357. 10.1002/wps.20255 26407793 PMC4592660

[r15] Dickson K, Ko SY(J), Nguyen C, Minchenko D and Bangpan M (2024) Mental health and psychosocial support programmes for displaced populations in low- and middle-income countries (LMICs): A systematic review of process, perspectives and experiences. Cambridge Prisms: Global Mental Health 11, e62. 10.1017/gmh.2024.56. 38774885 PMC11106547

[r16] Drescher A, Kiselev N, Akhtar A, Acarturk C, Bryant RA, Ilkkursun Z, von Känel R, Miller KE, Pfaltz MC, Schick M, Schnyder U, Sijbrandij M, Spaaij J and Morina N (2021) Problems after flight: Understanding and comparing Syrians’ perspectives in the Middle East and Europe. BMC Public Health 21 (1), 717. 10.1186/s12889-021-10498-1 33849507 PMC8045311

[r17] Echeverri C, Le Roy J, Worku B and Ventevogel P (2018) Mental health capacity building in refugee primary health care settings in sub-Saharan Africa: Impact, challenges and gaps. Global Mental Health 5, e28. 10.1017/gmh.2018.19 30202535 PMC6128042

[r18] Ekblad S, Gramatik O and Suprun Y (2024) Increasing perceived health and mental health literacy among separated refugee Ukrainian families with urgent needs occasioned by invasion-a group intervention study with participatory methodology in Sweden. Frontiers in Public Health 12, 1356605. 10.3389/fpubh.2024.1356605 38799690 PMC11122463

[r19] Elnasseh A, Mehta VS, Manolova G, Pedersen GA, Golden S, Eloul L, Gebrekristos F, Collins PY, Mutavi T, Mbwayo AW, Mathai M, Concepcion T, Masri RE, Steen F, Galea JT, Contreras C, Akellot J, Kasujja R, Wasereka S, Mutamba BB, Tol WA, Raji M, Moufarrej S, Schafer A and Kohrt BA (2024) Perspectives on competency-based feedback for training non-specialists to deliver psychological interventions: Multi-site qualitative study of the EQUIP competency-based approach. BJPsych Open 10 (4), e125. 10.1192/bjo.2024.37 38826043 PMC11363075

[r20] Epping-Jordan JE, Harris R, Brown FL, Carswell K, Foley C, García-Moreno C, Kogan C and van Ommeren M (2016) Self-help plus (SH+): A new WHO stress management package. World Psychiatry 15 (3), 295–296. 10.1002/wps.20355 27717271 PMC5032500

[r21] Frankova I, Vermetten E, Shalev AY, Sijbrandij M, Holmes EA, Ursano R, Schmidt U and Zohar J (2022) Digital psychological first aid for Ukraine. The Lancet Psychiatry 9 (7), e33. 10.1016/S2215-0366(22)00147-X 35526557

[r22] Goto R, Pinchuk I, Kolodezhny O, Pimenova N, Kano Y and Skokauskas N (2024) Mental health of adolescents exposed to the war in Ukraine. JAMA Pediatrics 178 (5), 480–488. 10.1001/jamapediatrics.2024.0295 38526470 PMC10964160

[r23] Goto R, Pinchuk I, Kolodezhny O, Pimenova N and Skokauskas N (2023) Mental health services in Ukraine during the early phases of the 2022 Russian invasion. The British Journal of Psychiatry 222 (2), 82–87. 10.1192/bjp.2022.170 36458514 PMC10964280

[r24] Guest G, Bunce A and Johnson L (2006) How many interviews are enough?: An experiment with data saturation and variability. Field Methods 18 (1), 59–82. 10.1177/1525822X05279903

[r25] Hennink MM, Kaiser BN and Marconi VC (2017) Code saturation versus meaning saturation: How many interviews are enough? Qualitative Health Research 27 (4), 591–608. 10.1177/1049732316665344 27670770 PMC9359070

[r26] Hollis C, Morriss R, Martin J, Amani S, Cotton R, Denis M and Lewis S (2015) Technological innovations in mental healthcare: Harnessing the digital revolution. The British Journal of Psychiatry: the Journal of Mental Science 206 (4), 263–265. 10.1192/bjp.bp.113.142612 25833865

[r27] Inter-Agency Standing Committee (IASC) (2007). IASC Guidelines on Mental Health and Psychosocial Support in Emergency Settings. Geneva: IASC. https://interagencystandingcommittee.org/sites/default/files/migrated/2020-11/IASC%20Guidelines%20on%20Mental%20Health%20and%20Psychosocial%20Support%20in%20Emergency%20Settings%20%28English%29.pdf 10.1080/09540261.2022.2147420 36502397

[r28] International Organization for Migration (IOM) (2024) Ukraine and Neighbouring Countries 2022–2024: Two Years of Response. https://www.iom.int/sites/g/files/tmzbdl486/files/documents/2024-02/iom_ukraine_neighbouring_countries_2022-2024_2_years_of_response.pdf.

[r29] Javanbakht A (2022) Addressing war trauma in Ukrainian refugees before it is too late. European Journal of Psychotraumatology 13 (2), 2104009. 10.1080/20008066.2022.2104009 35959204 PMC9359191

[r30] Javed A, Lee C, Zakaria H, Buenaventura RD, Cetkovich-Bakmas M, Duailibi K, Ng B, Ramy H, Saha G, Arifeen S, Elorza PM, Ratnasingham P and Azeem MW (2021) Reducing the stigma of mental health disorders with a focus on low- and middle-income countries. Asian Journal of Psychiatry 58, 102601. 10.1016/j.ajp.2021.102601 33611083

[r31] Jordan K, Lewis TP and Roberts B (2021) Quality in crisis: A systematic review of the quality of health systems in humanitarian settings. Conflict and Health 15 (1), 7. 10.1186/s13031-021-00342-z 33531065 PMC7851932

[r32] Keddem S (2021) Practical guidance for studies using Freelisting interviews. Preventing Chronic Disease 18. 10.5888/pcd17.200355 PMC7845553 33444525

[r33] Kerr C, Nixon A and Wild D (2010) Assessing and demonstrating data saturation in qualitative inquiry supporting patient-reported outcomes research. Expert Review of Pharmacoeconomics & Outcomes Research 10 (3), 269–281. 10.1586/erp.10.30 20545592

[r34] Koesters M, Barbui C and Purgato M (2018) Recent approaches to provision of mental healthcare in refugee populations. Current Opinion in Psychiatry 31 (4), 368–372. 10.1097/YCO.0000000000000428 29708893

[r35] Leask CF, Sandlund M, Skelton DA, Altenburg TM, Cardon G, Chinapaw MJM, De Bourdeaudhuij I, Verloigne M, Chastin SFM, and GrandStand, Safe Step and Teenage Girls on the Move Research Groups (2019) Framework, principles and recommendations for utilising participatory methodologies in the co-creation and evaluation of public health interventions. Research Involvement and Engagement 5, 2. 10.1186/s40900-018-0136-9. 30652027 PMC6327557

[r36] Lotito C, Turrini G, Purgato M, Bryant RA, Felez-Nobrega M, Haro JM, Lorant V, McDaid D, Mediavilla R, Melchior M, Nicaise P, Nosè M, Park A-L, McGreevy KR, Roos R, Tortelli A, Underhill J, Martinez JV, Witteveen A, Sijbrandij M and Barbui C (2023) Views and experiences of migrants and stakeholders involved in social and health care for migrants in Italy during the COVID-19 pandemic: A qualitative study. BMC Psychology 11, 164. 10.1186/s40359-023-01208-0 37208725 PMC10198022

[r37] Lund C, Brooke-Sumner C, Baingana F, Baron EC, Breuer E, Chandra P, Haushofer J, Herrman H, Jordans M, Kieling C, Medina-Mora ME, Morgan E, Omigbodun O, Tol W, Patel V and Saxena S (2018) Social determinants of mental disorders and the sustainable development goals: A systematic review of reviews. The Lancet. Psychiatry 5 (4), 357–369. 10.1016/S2215-0366(18)30060-9 29580610

[r38] Mabil-Atem JM, Gumuskaya O and Wilson RL (2024) Digital mental health interventions for the mental health care of refugees and asylum seekers: Integrative literature review. International Journal of Mental Health Nursing 00, 1–21. 10.1111/inm.13283 38291740

[r39] Mediavilla R, Monistrol-Mula A, McGreevy KR, Felez-Nobrega M, Delaire A, Nicaise P, Palomo-Conti S, Bayón C, Bravo-Ortiz M-F, Rodríguez-Vega B, Witteveen A, Sijbrandij M, Turrini G, Purgato M, Vuillermoz C, Melchior M, Petri-Romão P, Stoffers-Winterling J, Bryant RA, McDaid D, Park A-L and Ayuso-Mateos JL (2022) Mental health problems and needs of frontline healthcare workers during the COVID-19 pandemic in Spain: A qualitative analysis. Frontiers in Public Health 10, 956403. 10.3389/fpubh.2022.956403 35968478 PMC9363705

[r40] Morse JM (1995) The significance of saturation. Qualitative Health Research 5 (2), 147–149. 10.1177/104973239500500201

[r41] Naderifar M, Goli H and Ghaljaie F (2017) Snowball sampling: A purposeful method of sampling in qualitative research. Strides in Development of Medical Education 14 (3). 10.5812/sdme.67670

[r42] Naeem M, Ozuem W, Howell K and Ranfagni S (2023) A step-by-step process of thematic analysis to develop a conceptual model in qualitative research. International Journal of Qualitative Methods 22, 16094069231205789. 10.1177/16094069231205789

[r43] Papola D, Prina E, Ceccarelli C, Cadorin C, Gastaldon C, Ferreira MC, Tol WA, van Ommeren M, Barbui C and Purgato M (2024) Psychological and social interventions for the promotion of mental health in people living in low- and middle-income countries affected by humanitarian crises. The Cochrane Database of Systematic Reviews 5(5), CD014300. 10.1002/14651858.CD014300.pub2. PMC11106803 38770799

[r44] Proctor E, Bunger AC, Lengnick-Hall R, Gerke DR, Martin JK, Phillips RJ and Swanson JC (2023) Ten Years of implementation outcomes research: A scoping review. Implementation Science 18(1), 31. 10.1186/s13012-023-01286-z. 37491242 PMC10367273

[r45] Proctor E, Silmere H, Raghavan R, Hovmand P, Aarons G, Bunger A, Griffey R and Hensley M (2011) Outcomes for implementation research: Conceptual distinctions, measurement challenges, and research agenda. Administration and Policy in Mental Health 38 (2), 65–76. 10.1007/s10488-010-0319-7 20957426 PMC3068522

[r46] Purgato M, Tol WA and Bass JK (2017) An ecological model for refugee mental health: Implications for research. Epidemiology and Psychiatric Sciences 26 (2), 139–141. 10.1017/S204579601600069X 27641739 PMC6998686

[r47] Rizzi D, Ciuffo G, Sandoli G, Mangiagalli M, de Angelis P, Scavuzzo G, Nych M, Landoni M and Ionio C (2022) Running away from the war in Ukraine: The impact on mental health of internally displaced persons (IDPs) and refugees in transit in Poland. International Journal of Environmental Research and Public Health 19 (24), 16439. 10.3390/ijerph192416439 PMC9778520 36554321

[r48] Romao P, Neuenschwander S, Denecke K and Nüssli S (2021) Digital health intervention to support refugees in Switzerland. Studies in Health Technology and Informatics 279, 95–102. 10.3233/SHTI210094 33965924

[r49] Schäfer SK, Thomas LM, Lindner S and Lieb K (2023) World Health Organization’s low-intensity psychosocial interventions: A systematic review and meta-analysis of the effects of problem management plus and step-by-step. World Psychiatry: Official Journal of the World Psychiatric Association (WPA) 22 (3), 449–462. 10.1002/wps.21129 37713578 PMC10503931

[r50] Seifert A, Reinwand DA and Schlomann A (2019) Designing and using digital mental health interventions for older adults: Being aware of digital inequality. Frontiers in Psychiatry 10, 568. 10.3389/fpsyt.2019.00568. 31447716 PMC6696744

[r51] Seleznova V, Pinchuk I, Feldman I, Virchenko V, Wang B and Skokauskas N (2023) The battle for mental well-being in Ukraine: Mental health crisis and economic aspects of mental health services in wartime. International Journal of Mental Health Systems 17 (1), 28. 10.1186/s13033-023-00598-3 37749608 PMC10518916

[r52] Speziale HS, Streubert HJ and Carpenter DR (2011) Qualitative Research in Nursing: Advancing the Humanistic Imperative. Lippincott Williams & Wilkins.

[r53] Torous J, Wisniewski H, Liu G and Keshavan M (2018) Mental health Mobile phone app usage, concerns, and benefits among psychiatric outpatients: Comparative survey study. JMIR Mental Health 5 (4), e11715. 10.2196/11715 30446484 PMC6269625

[r54] Troup J, Fuhr DC, Woodward A, Sondorp E and Roberts B (2021) Barriers and facilitators for scaling up mental health and psychosocial support interventions in low- and middle-income countries for populations affected by humanitarian crises: A systematic review. International Journal of Mental Health Systems 15 (1), 5. 10.1186/s13033-020-00431-1 33413526 PMC7792016

[r55] Turrini G, Purgato M, Ballette F, Nosè M, Ostuzzi G and Barbui C (2017) Common mental disorders in asylum seekers and refugees: Umbrella review of prevalence and intervention studies. International Journal of Mental Health Systems 11 (1), 51. 10.1186/s13033-017-0156-0 28855963 PMC5571637

[r56] Turrini G, Tedeschi F, Cuijpers P, Del Giovane C, Kip A, Morina N, Nosè M, Ostuzzi G, Purgato M, Ricciardi C, Sijbrandij M, Tol W and Barbui C (2021) A network meta-analysis of psychosocial interventions for refugees and asylum seekers with PTSD. BMJ Global Health 6 (6), e005029. 10.1136/bmjgh-2021-005029 PMC8183228 34088735

[r57] United Nations High Commissioner for Refugees (UNHCR) (2024) Situation Ukraine Refugee. https://data.unhcr.org/en/situations/ukraine (accessed 24 May 2024).

[r58] Vitruk O (2023) Ukrainian Refugee Mental Health Profile. https://ethnomed.org/resource/ukrainian-refugee-mental-health-profile/ (accessed 24 May 2024).

[r59] World Health Organization (WHO) (2013) Building Back Better: Sustainable Mental Health Care after Emergencies: Overview. Geneva: WHO. https://iris.who.int/handle/10665/96378

[r60] World Health Organization (WHO) (2022) World Mental Health Report: Transforming Mental Health for All. Geneva: WHO. https://iris.who.int/bitstream/handle/10665/356119/9789240049338-eng.pdf?sequence=1

[r61] World Health Organization (WHO) (2023a) Global Research Agenda on Health, Migration and Displacement: Strengthening Research and Translating Research Priorities into Policy and Practice. Geneva: WHO. https://iris.who.int/bitstream/handle/10665/373659/9789240082397-eng.pdf?sequence=1

[r62] World Health Organization (WHO) (2023b) Mental health of refugees and migrants: Risk and protective factors and access to care. In Global Evidence Review on Health and Migration (GEHM) Series. Geneva: WHO. https://iris.who.int/bitstream/handle/10665/373279/9789240081840-eng.pdf?sequence=1 37972226

[r63] World Health Organization (WHO) (2024) Psychological Interventions Implementation Manual: Integrating Evidence-Based Psychological Interventions into Existing Services. Geneva: WHO. https://iris.who.int/bitstream/handle/10665/376208/9789240087149-eng.pdf?sequence=1

[r64] World Health Organization (WHO) and United Nations High Commissioner for Refugees (UNHCR) (2012) Assessing Mental Health and Psychosocial Needs and Resources: Toolkit for Humanitarian Settings. Geneva: WHO. https://iris.who.int/handle/10665/76796

[r65] World Health Organization (WHO), War Trauma Foundation (WTF) and World Vision International (WVI) (2011) Psychological First Aid: Guide for Field Workers. Geneva: WHO. https://iris.who.int/bitstream/handle/10665/44615/9789241548205_eng.pdf?sequence=1

