# Supplementary material for: Assessing local service providers’ needs for scaling up MHPSS interventions for Ukrainian refugees: Insights from Poland, Slovakia, and Romania
Source: Glob Ment Health (Camb). 2024 Dec 6;11:e119. doi: 10.1017/gmh.2024.113 (PMC11704377; doi:10.1017/gmh.2024.113)
Supplement: Purgato et al. supplementary material [file S2054425124001134sup001.docx]

**Appendix**

**Supplementary material**

**Summary**

1. Quotes from Free Listing and Key Informant Interviews 1
2. **Table 1S.** Recommended actions for addressing barriers to implementing in-person MHPSS interventions 5
3. **Table 2S.** Challenges in Digital MHPSS Implementation: Generational Obstacles, Therapeutic Relationship, Trust, and Awareness 9
4. **Table 3S.** Recommended actions for addressing barriers to implementing digital

MHPSS 12

1. **Table 4S.** Implementation model of Proctor and socio-ecological framework of Bronfenbrenner 15
2. **Figure 1S.** Identified challenges and barriers 18
3. **Figure 2S.** Barriers linked to the socio-ecological framework

of Bronfenbrenner 20

**Quotes from Free Listing and Key Informant Interviews**

**Implementation barriers to in-person psychosocial interventions: nature of the problem, causes, and consequences**

***Stigma***

*The stigma around getting mental health services is very high. (…) there was a big reluctance to seek mental health services because people feared that they would be labeled as weak and because they could not manage to solve their own problems.” (KIPS3)*

*“When we were doing the need assessment, in the beginning, they refused, they were not open to discussing the challenges they are facing while living in the refugee centers or living in the community.”* (KIPS3)

***Language barriers***

*"It's actually very interesting because, for some people, it means a lack of a job, while for others, it means having too much work. For instance, those working on projects providing psychological help to refugees may find themselves with an overwhelming number of clients. So, there are essentially two very different sides to the same problem." (KIPS2).*

***Lack of MHPSS professionals and infrastructure***

*“Clinics are overloaded, with even psychiatrists facing heavy caseloads. For instance, when a Slovak citizen experiences a mental health issue, the wait time for assistance is a minimum of three months. The situation is even more challenging for Ukrainians* (…)*. We see a huge shortage of mental health professionals and infrastructure, ranging from basic peer support to licensed professionals and even inpatient psychiatric treatment facilities.”* (KISS4)

*“The second is the difficulty of this profession. Because there are often cases where people can burn out very quickly. The work is very demanding, there are various difficult, complex problems that need to be solved and thus, it is problematic to retain such people for such a low salary.”*

***Financial barriers***

*“From our perspective, it's one of the most crucial services, as it helps individuals cope with mental health challenges, enabling them to address various other issues such as integration, finding employment, performing well at their jobs, and taking care of their families. However* (…) *there seems to be a prevailing sentiment that there are always more pressing priorities”* (KIPS5)*.*

*“Writing projects can be complicated because you need specialized individuals who can do it, or you need to pay them. This can be challenging for your organization or your employees if they lack the time or knowledge to write projects.”*

***Trust barriers***

*“It is a general sense of distrust, possibly from past experiences, especially for those who lived in the Soviet Union. This is just my opinion, but it seems to be a pervasive feeling of mistrust towards everything, including other people offering help. Building trust takes time, and we need to approach each person in a special way to earn their trust.”* (KISS 1)

**Implementation barriers to digital psychosocial interventions***:* **problem, causes and consequences**

*“They are afraid to make a mistake in a certain way, to press an incorrect button. If they do something wrong, they don’t know how to correct it, and they become vulnerable because they must ask for help again and again.”* (KIRS4)

*“The main challenge for those who want to deliver such applications is that they invest a lot of time and effort into it. However, they often struggle to attract users to the app. Without a user base, all the work put into developing the app feels like wasted effort. Additionally, receiving feedback from users is crucial for improving the app, but without users, this feedback is lacking”.* (KIRS4)

*“I believe these interventions are not widely discussed or promoted. For instance, it was a revelation for me to learn about interventions like Self-Help Plus being available online. I had no idea until the trainer informed me about it during their visit.”* (KIPS2)

*“For service providers, such tools can assist them in alleviating their workload. Not every client necessarily requires extensive therapy; some may benefit from simple tools that empower them to help themselves effectively.”* (KIPS2)

**Table 1S.** Recommended actions for addressing barriers to implementing in-person MHPSS interventions.

| **Recommendations** | **Level of intervention** | **Addressed Barriers** |
| --- | --- | --- |
| **ADVANCING PUBLIC HEALTH STRATEGIES** | | |
| - Developing state-level policy for organizing MHPSS responses | Public Policy, Organization | Stigma, lack of MHPSS professionals, financial barriers, trust barriers |
| - Creating a comprehensive national plan that outlines guidelines for crisis intervention at the national, regional, and local levels. | Public Policy and Organization |  |
| - Developing National level scaling up strategies | Public Policy, Organization |  |
| - Providing state-reimbursed MHPSS services (public health approach) | Public Policy |  |
| - State-level consistent and reliable actions and policies to ensure long-term predictability. | Public Policy |  |
| **ENHANCING FUNDING OPPORTUNITIES AND ACCESSIBILITY** | | |
| - Financial support for MHPSS activities | Public Policy | Stigma, financial barriers |
| - Mobilization of financial resources through donor support. | Organizations |  |
| - Encouraging organizations to apply for EU funds | Public Policy |  |
| - Create training and support for organizations and NGOs to efficiently access and manage funds | Organizations |  |
| **INCORPORATING UKRAINIAN SPECIALISTS IN ORGANIZATIONS AND PROJECTS PROVIDING MHPSS** | | |
| - Incorporating Ukrainian specialists into the mental healthcare system of the host country | Public Policy | Language barriers, lack of MHPSS professionals, financial barriers |
| - Creating simplified procedures for the recognition of academic studies and certificates of Ukrainian MHPSS workers | Public Policy |  |
| - Advocacy for enabling Ukrainian mental health professionals to serve their own community | Organizations, Individual providers |  |
| - Engaging Ukrainian MHPSS specialists in translation activities | Organizations, Individual providers |  |
| **STRENGTHENING COLLABORATIVE AND COORDINATED MHPSS RESPONSES** | | |
| - Collaboration among government entities, NGOs, social workers, and mental health specialists to deliver coordinated psychosocial interventions | Public Policy, Organization | Language barriers, lack of MHPSS professionals, financial barriers, trust barriers |
| - Establishing clear referral pathways between organizations and specialists for smoother coordination | Public Policy, Organization |  |
| - Collaboration between NGOs to maximize human resources | Organizations |  |
| - Conducting needs assessment at different stages of the conflict | Public Policy, Organization |  |
| - Collaboration among NGOs, state and municipalities | Public Policy, Organization |  |
| - Engaging interpreters into MHPSS activities | Organizations |  |
| - Create training for organizations to efficiently organize large-scale MHPSS | Organizations |  |
| **PSYCHOEDUCATION AND MENTAL HEALTH AWARENESS INITIATIVES** | | |
| - Organizing psychoeducation awareness campaigns about mental health problems and services | Public Policy, Organization | Stigma, financial barriers, trust barriers |
| - Ensure social media presence and relevant content on MHPSS topics | Organizations |  |
| **FOSTERING GROUP AND COMMUNITY-BASED ACTIVITIES** | | |
| - Family-based programs for developing parenting skills | Organizations | Stigma, language barriers, lack of MHPSS professionals, financial barriers, trust barriers |
| - Delivering group format Psychosocial interventions | Organizations, Individual providers |  |
| - Providing community-based psychosocial activities | Organizations, Individual providers |  |
| - Providing community-based recreational and cultural activities | Public Policy, Organizations, Individual providers |  |
| - Providing joint community activities where Ukrainians and host populations can interact | Public Policy, Organizations, Individual providers |  |
| **TRAINING INITIATIVES FOR MHPSS SPECIALISTS** | | |
| - Training for MHPSS professionals to overcome stigma | Organizations, Individual providers | Stigma, trust barriers |
| - Support MHPSS specialists through training, intervision and supervision | Organizations, Individual providers |  |
| **PROMOTION OF DIGITAL MHPSS** | | |
| - Delivering online MHPSS | Organizations, Individual providers | Stigma, lack of MHPSS professionals, financial barriers |
| **TRAINING INITIATIVES FOR HELPERS AND LAY WORKERS** | | |
| - Training key community figures to build psychosocial competencies | Organizations | Stigma, trust barriers, lack of MHPSS professionals |
| - Providing formal accredited training programs for MHPSS helpers and lay workers | Organizations |  |
| - Engaging volunteers in mental health initiatives. | Organizations |  |
| **LANGUAGE EDUCATION INITIATIVES FOR UKRAINIAN REFUGEES** | | |
| - Free language courses for Ukrainian refugees | Public Policy, Organization | Language barriers |
| - Encourage Ukrainians to learn the local language | Public Policy, Organizations,  Individual providers |  |

**Table 2S.** Challenges in Digital MHPSS Implementation: Generational Obstacles, Therapeutic Relationship, Trust, and Awareness.

The logic pathway to link responses to the theoretical framework reported here and in Table 4S relates to the impact of the specific responses on implementation outcomes and on the social and psychological effects according to the socio-ecological model. Links have been established based on the literature in small-group meetings with expert researchers of this authors’ team.

| **Barrier** | **Implementation outcome**  **(Proctor, 2011)** | **Level** | **Cause** | **Implementation outcome effect (Proctor, 2011)** | **Level** | **Effect** |
| --- | --- | --- | --- | --- | --- | --- |
| Generational obstacles | Acceptability and appropriateness | Individual consumer | Technical illiteracy | Adoption, feasibility, penetration, and sustainability | Individual providers and organization | Inability to reach and engage older Ukrainian refugees with digital MHPSS |
|  |  |  | Use of old technology |  | Individual consumer, Individual providers and organization | Lack of uptake |
|  |  |  | Lack of self-efficacy |  | Individual providers and organization | Lack of development of digital solutions |
|  |  | Individual consumer, organization | Novelty of technology |  | Individual providers and organization | Lack of promotion of digital interventions |
|  |  |  | Novelty of digital interventions |  |  |  |
|  |  | Individual consumer, individual provider | Perceived lack of efficacy |  |  |  |
| Lack of therapeutic relationship |  |  | SP and UR cultural perception of MHPSS |  |  |  |
|  |  |  | The severe nature of the social determinant |  |  |  |
| Lack of trust |  |  | SP and UR safety concerns |  |  |  |
|  |  |  | SP and UR data security concerns |  |  |  |
|  |  |  | SP and UR perceived lack of evidence |  |  |  |
|  |  |  | SP and UR perceived lack of efficacy |  |  |  |
|  |  |  | SP and UR perceived low-quality design |  |  |  |
|  |  |  | SP and UR perceived low-quality translation to Ukrainian |  |  |  |
| Lack of awareness |  | Individual consumer, Individual providers, organization | Lack of promotion |  |  |  |

**Table 3S.** Recommended actions for addressing barriers to implementing digital MHPSS.

| **Recommendation** | **Level of intervention** | **Addressed Barries** |
| --- | --- | --- |
| **ADVANCING PUBLIC HEALTH STRATEGIES FOR PROMOTING DIGITAL MHPSS** | | |
| - Develop a comprehensive national plan for the seamless integration of digital interventions into mental health services. | Public policy, organizations, individual providers | Trust, awareness |
| **BUILDING TECHNICAL LITERACY AND SUPPORT** | | |
| - Providing education on the utilization of digital interventions | Public policy, organizations, individual providers | Generational |
| - Supplying personal technical support to facilitate adoption | Organizations, individual providers |  |
| - Engage family members to increase technical literacy | Organizations, individual providers |  |
| - Ensuring access to appropriate technology for the elderly to engage with digital interventions | Public policy, organizations |  |
| **BUILDING CREDIBILITY** | | |
| - Conducting efficacy research | Public policy, organizations | Trust, Awareness |
| - Disseminating efficacy research | Public policy, organizations, individual providers |  |
| - Endorsement by researchers and medical staff | Public policy, organizations |  |
| **TESTING ACCEPTABILITY AND PRODUCT QUALITY** | | |
| - Creating focus groups with beneficiaries | Organizations, individual providers | Trust, Awareness |
| - Field testing and feedback by beneficiaries | Organizations, individual providers |  |
| **TRAINING INITIATIVES FOR MHPSS WORKERS** | | |
| - Providing training on digital interventions to MHPSS professionals | Organizations | Trust, Awareness |
| - Organizing conferences and webinars for MH specialists | Organizations |  |
| **FOSTERING AWARENESS AND DISSEMINATION** | | |
| - Promoting awareness about the benefits and usage of digital tools | Public policy, organizations, individual providers | Generational, Trust, Awareness |
| - Endorsement by trusted members of the community | Organizations |  |
| - Advertising by Ukrainian refugees | Public policy, organizations |  |
| - Advocacy by NGOs and MHPSS professionals | Organizations, individual providers |  |
| - Ethical marketing | Public policy, organizations, individual providers |  |
| - Marketed as a tool for helping loved ones and building reliance | Public policy, organizations, individual providers |  |
| - Promote digital interventions in locations where Ukrainian refugees frequently congregate | Public policy, organizations, individual providers |  |
| **INCORPORATION OF DIGITAL INTERVENTIONS INTO USUAL PRACTICE** | | |
| - Introducing digital interventions gradually, starting with in-person MHPSS | Public policy, organizations, individual providers | Generational, Lack of therapeutic relationship, trust |
| - Integration of digital tools in in-person therapy (hybrid interventions) | Organizations, individual providers |  |
| - Gradually introducing digital interventions into MHPSS | Organizations, individual providers |  |
| - Integrate digital intervention with organizations that work with Ukrainian refugees on various psychosocial problems | Organizations, individual providers |  |
| - Using digital interventions with outpatients | Organizations, individual providers |  |

**Table 4S.** Implementation model of Proctor and socio-ecological framework of Bronfenbrenner.

| **Theme** | **Implementation outcomes causes** | **Cause** | **Implementation outcomes effects** | **Effects according to impementation model (Proctor)** | **Social-psychological effects according to the theory of Brofenbrenner** |
| --- | --- | --- | --- | --- | --- |
| Generational obstacles | Acceptability and appropriateness, feasibility | Technical illiteracy, Use of old technology | Adoption, feasibility, penetration and sustainability | Inability to reach and engage older Ukrainian refugees with digital MHPSS |  |
|  | Acceptability and appropriateness | Novelty of digital interventions, Novelty of technology | Adoption, penetration and sustainability | Inability to reach and engage older Ukrainian refugees with digital MHPSS, | Isolation and vulnerability of older Ukrainian refugees, Untreated of mental health problems in older Ukrainian refugees |
|  | Acceptability and appropriateness | Lack of therapeutic relationship | Adoption and feasibility | Inability to reach and engage older Ukrainian refugees with digital MHPSS, | Isolation and vulnerability of older Ukrainian refugees, Untreated of mental health problems in older Ukrainian refugees |
|  | Acceptability and appropriateness | Perceived lack of efficacy | Adoption, penetration and sustainability | Inability to reach and engage older Ukrainian refugees with digital MHPSS, | Isolation and vulnerability of older Ukrainian refugees, Untreated of mental health problems in older Ukrainian refugees |
|  | Acceptability and appropriateness | Lack of self-efficacy | Adoption and feasibility | Inability to reach and engage older Ukrainian refugees with digital MHPSS, | Isolation and vulnerability of older Ukrainian refugees, Untreated of mental health problems in older Ukrainian refugees |
| Lack of therapeutic relationship | Acceptability and appropriateness | SP and UR cultural perception about MHPSS | Adoption, feasibility and sustainability | Lack of uptake, lack of development, lack of promotion | Lack of trust |
|  | Acceptability and Appropriateness | Sever nature of the social determinant | Adoption, feasibility, penetration | Lack of uptake, lack of development, lack of promotion | Lack of trust |
| Lack of trust | Acceptability and appropriateness | Lack of therapeutic relationship | Adoption, feasibility, penetration | Lack of uptake, lack of development, lack of promotion |  |
|  | Acceptability and appropriateness | SP and UR safety concern | Adoption, penetration, sustainability | Lack of uptake, lack of development, lack of promotion |  |
|  | Acceptability and appropriateness | SP and UR data security concern | Adoption, penetration, sustainability | Lack of uptake, lack of development, lack of promotion |  |
|  | Acceptability, and appropriateness | SP and UR perceived lack of evidence | Adoption, penetration and sustainability | Lack of uptake, lack of development, lack of promotion |  |
|  | Acceptability, and appropriateness | SP and UR perceived lack of efficacy | Adoption, penetration and sustainability | Lack of uptake, lack of development, lack of promotion |  |
|  | Acceptability and appropriateness | SP and UR perceived low quality design | Adoption , penetration and sustainability | Lack of uptake, lack of development, lack of promotion |  |
|  | Acceptability and appropriateness | SP and UR perceived low quality translatin to ukrainian | Adoption penetration and sustainability | Lack of uptake, lack of development, lack of promotion |  |
| Lack of awareness | Acceptability and appropriateness | Lack of promotion | Adoption, feasibility, implementation cost, penetration, sustainability | burden of MH specialists | Lack of trust, Mental health problems and dysfunctional coping mechanisms transmitted across generations, Social impairment, Medical conditions, Unhealthy coping mechanisms, Untreatted and aggravated mental health problems |


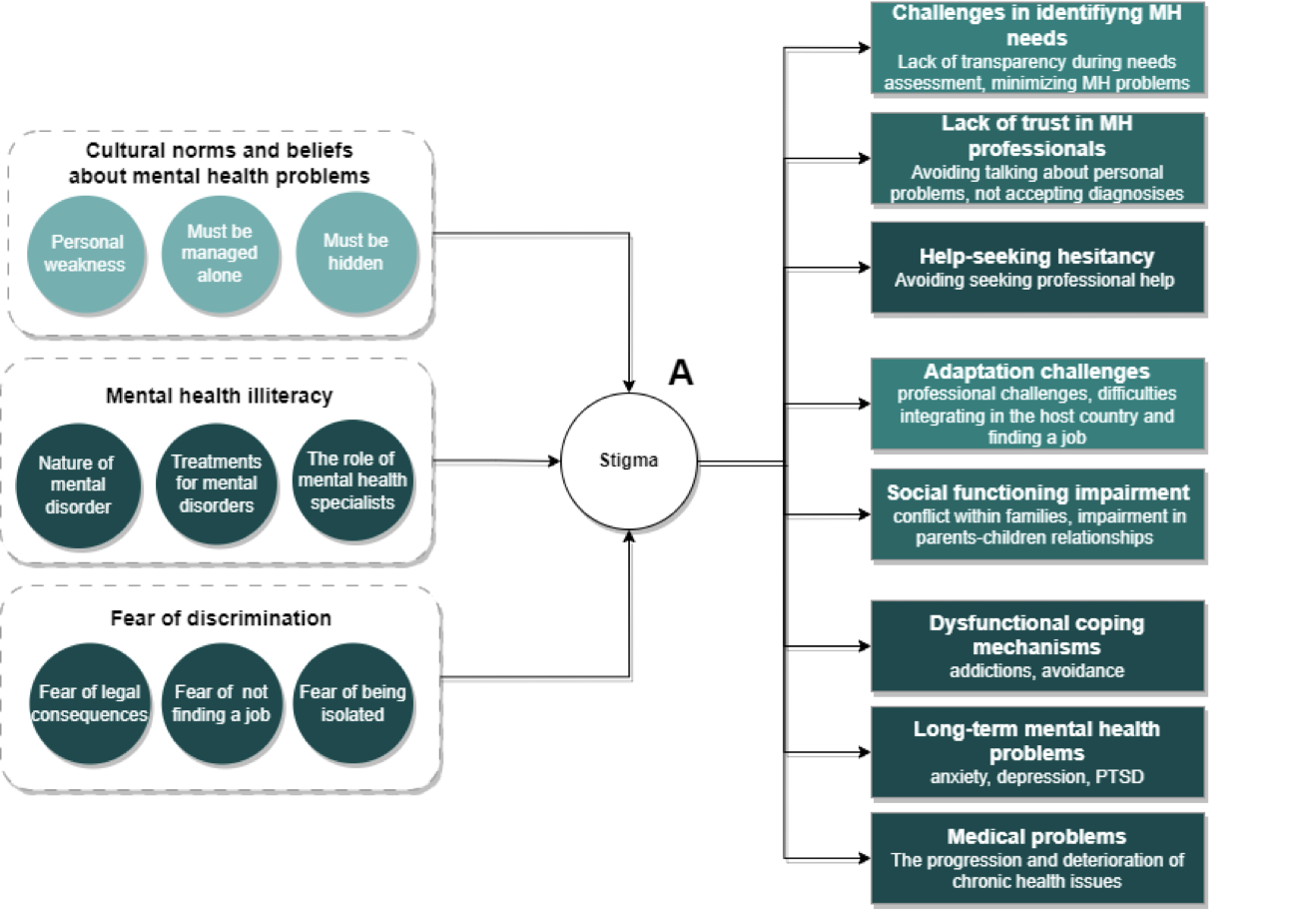

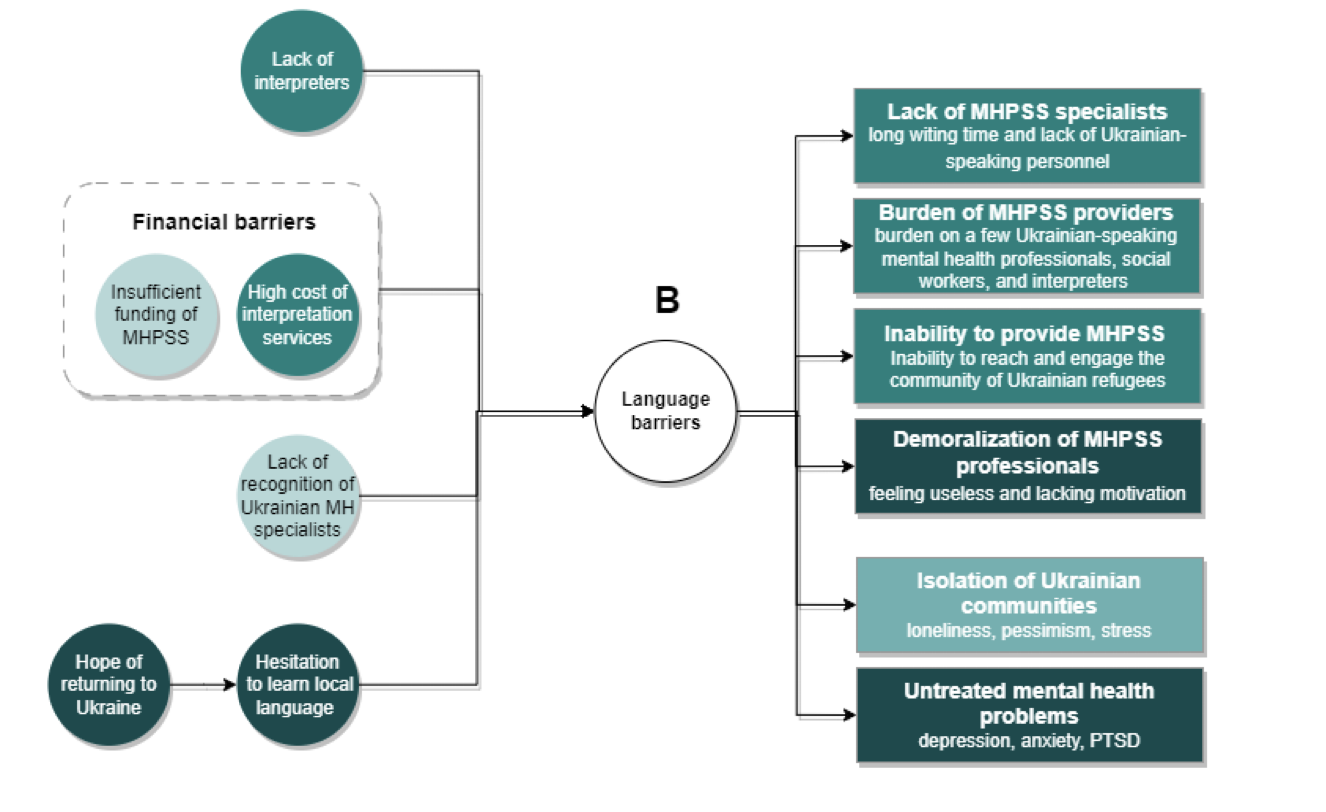


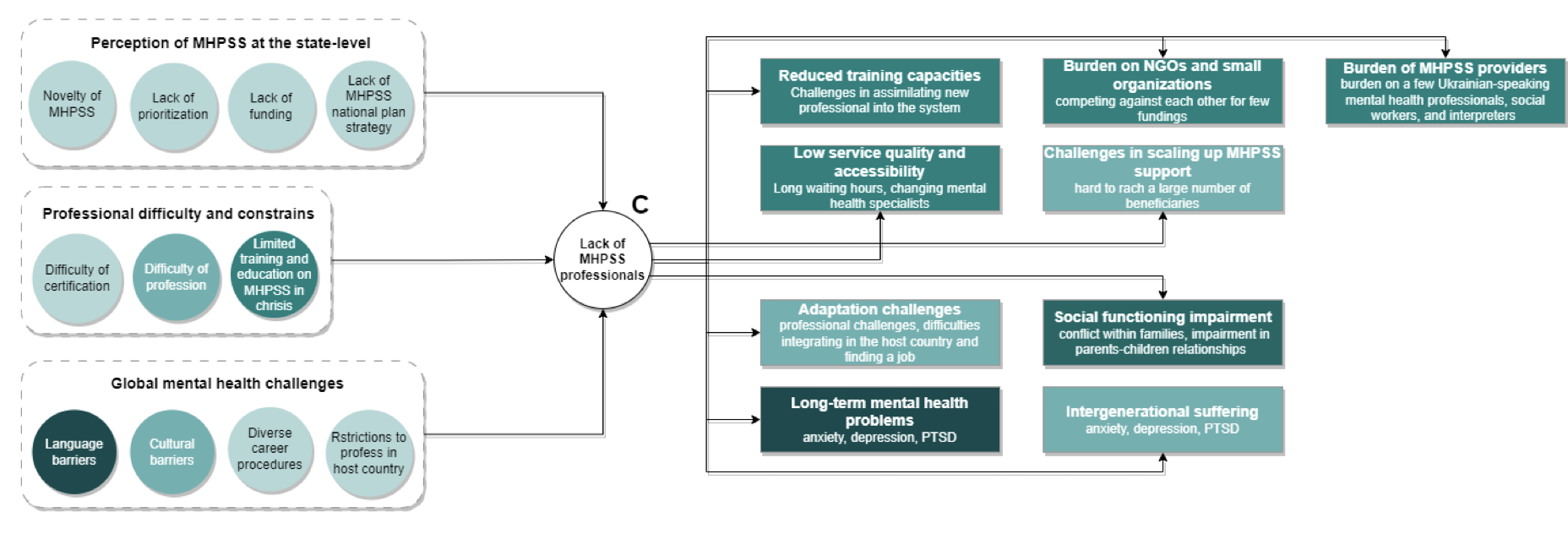


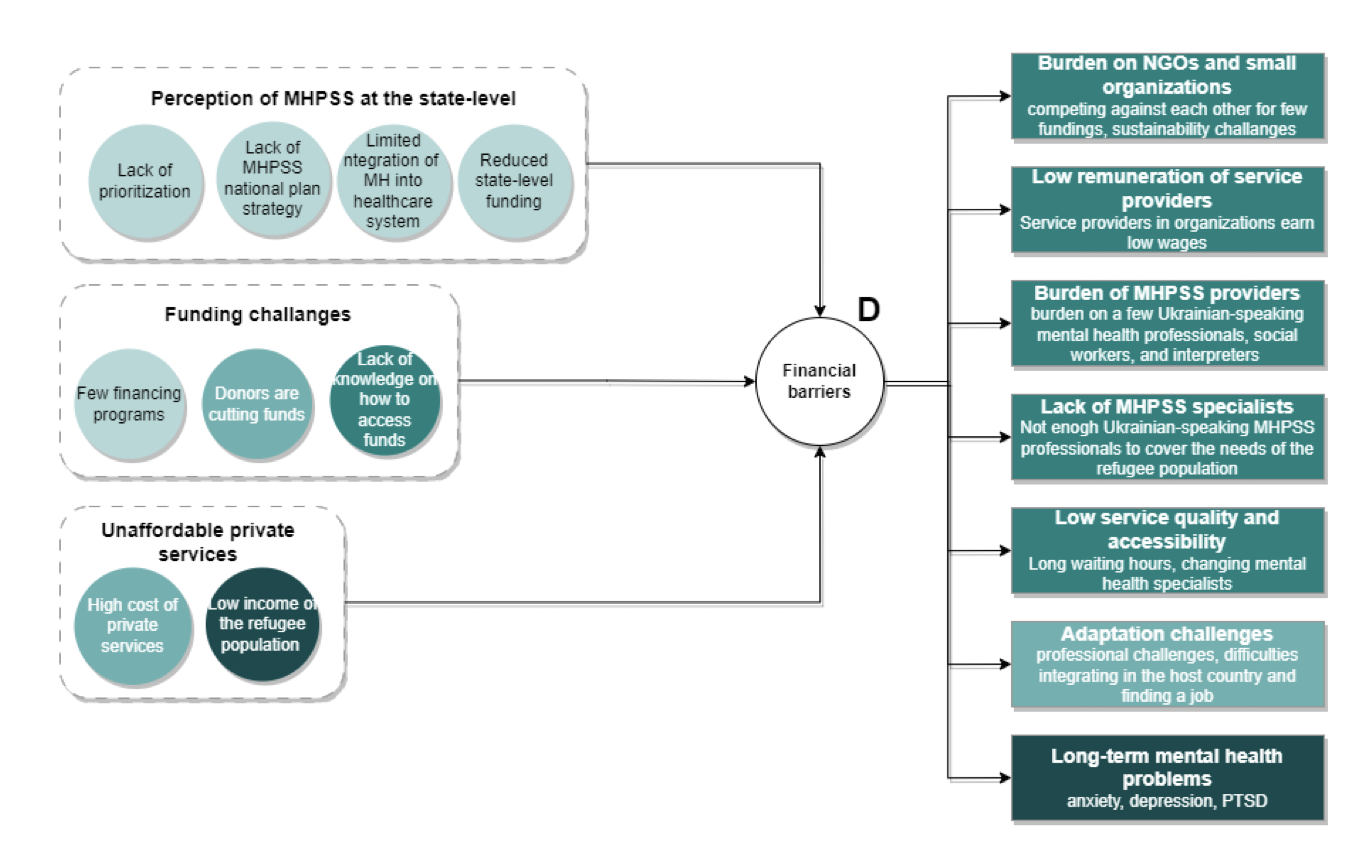


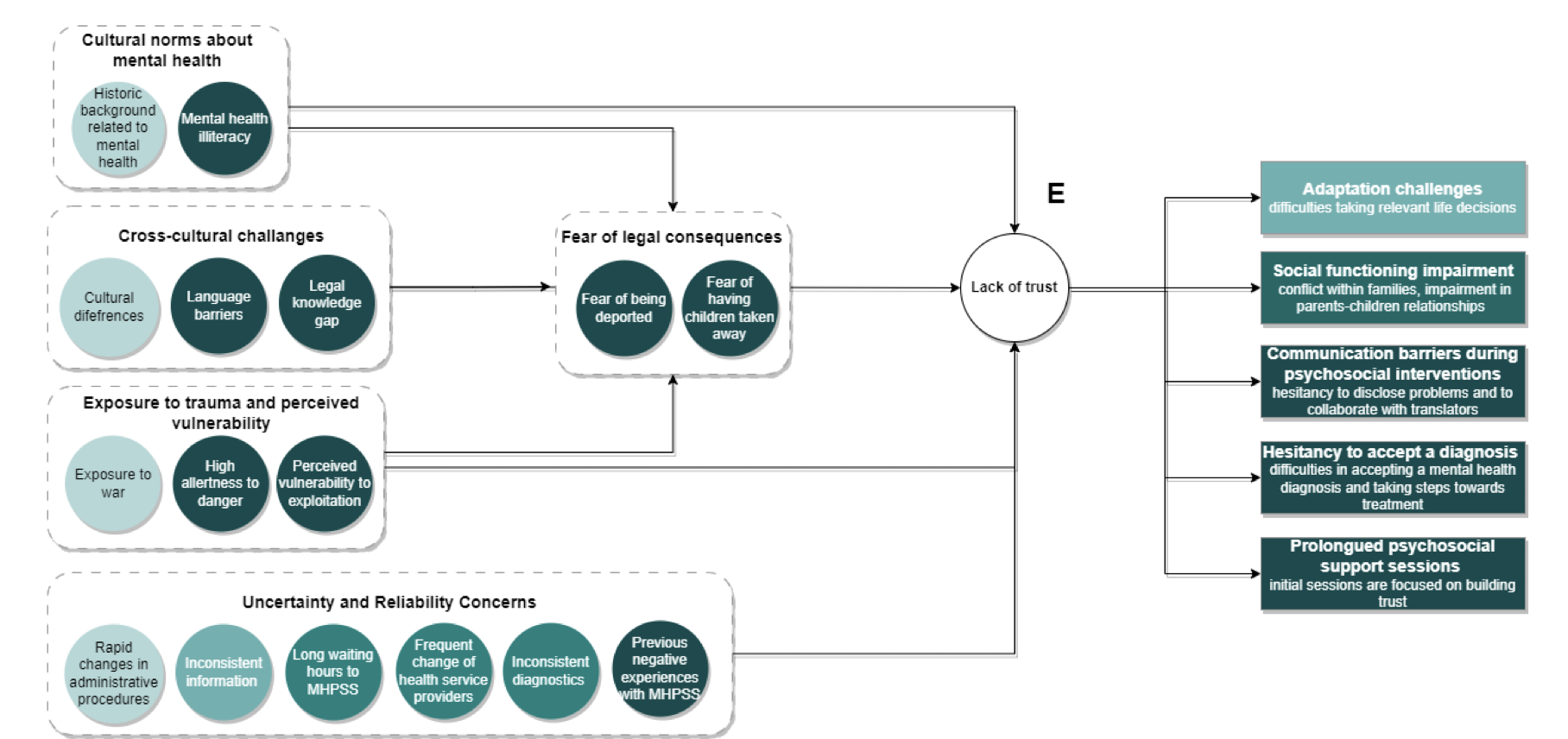


**Figure 1S.** Identified challenges and barriers.


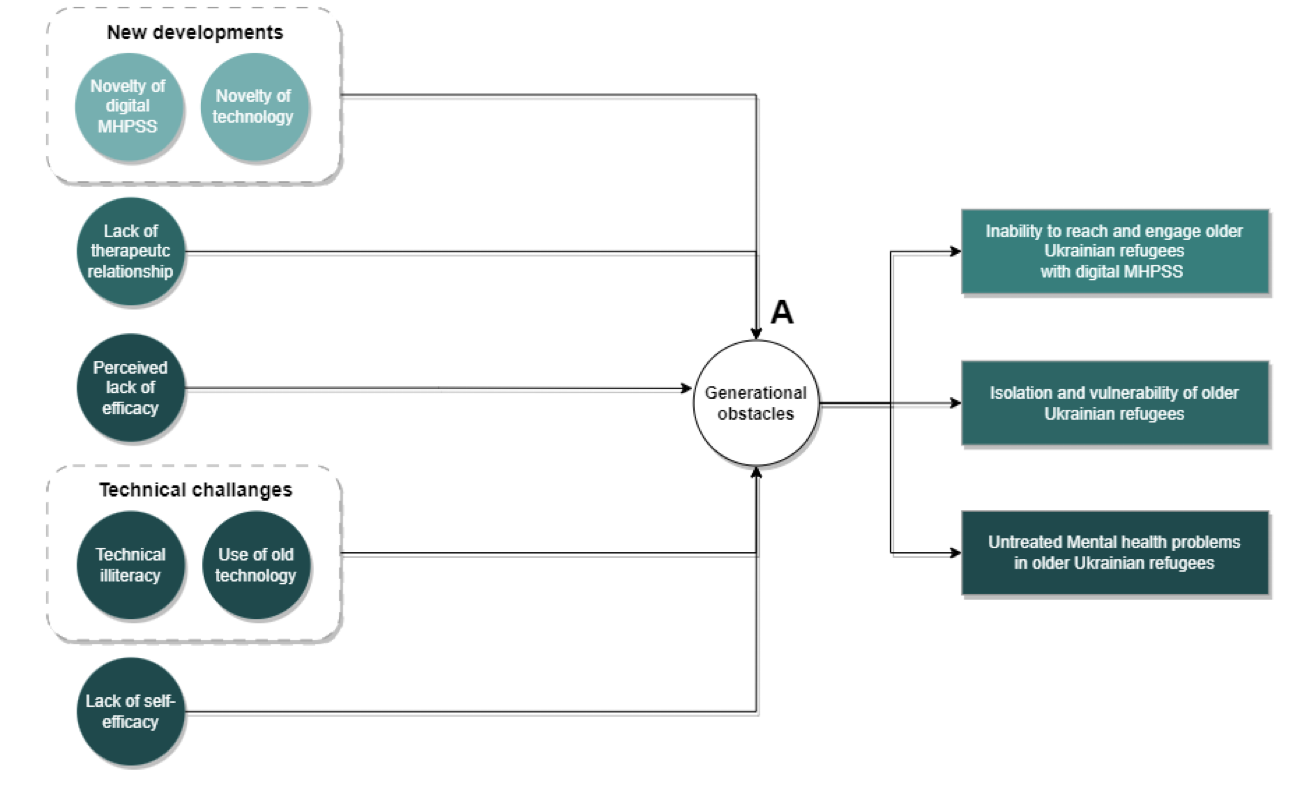


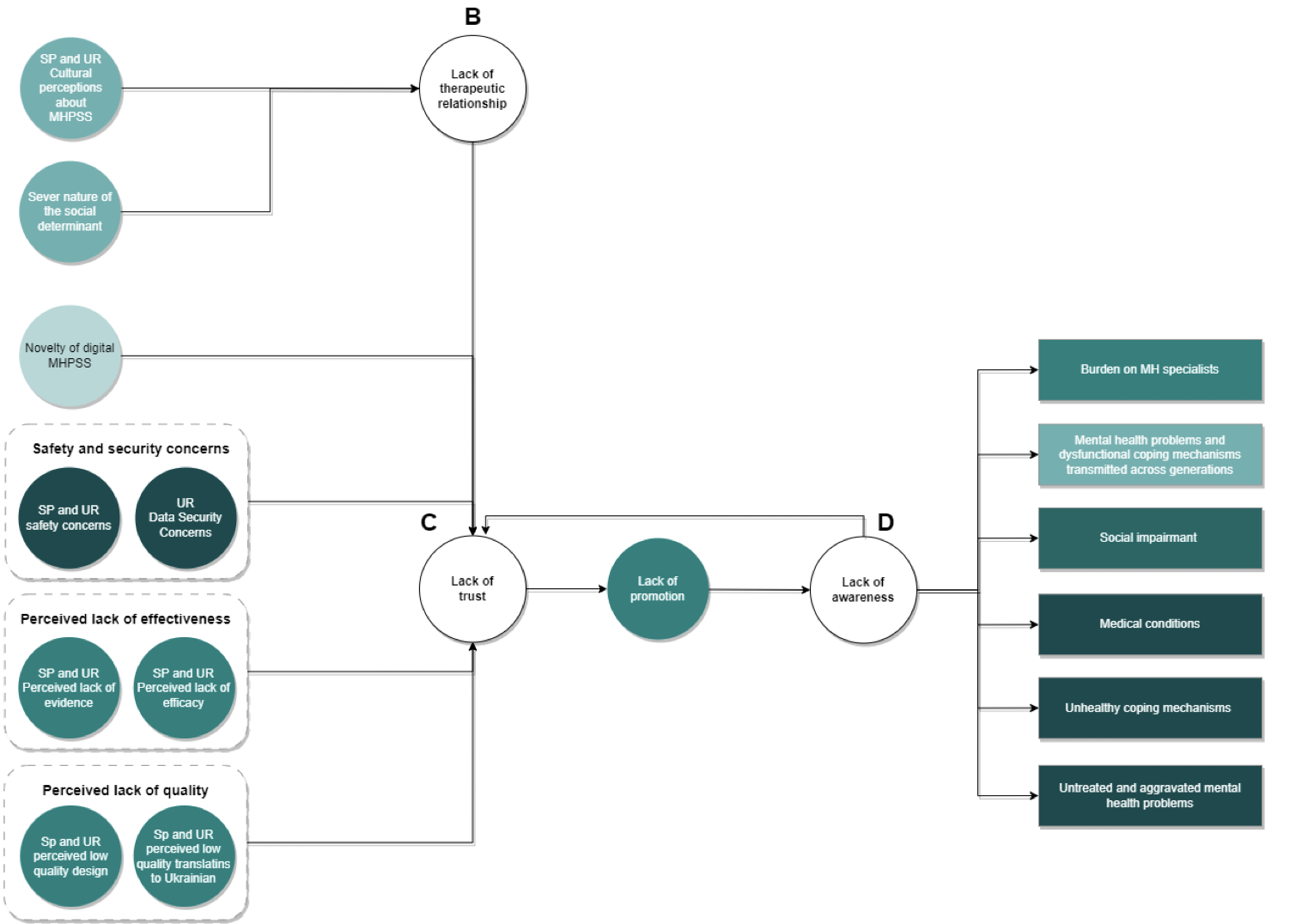


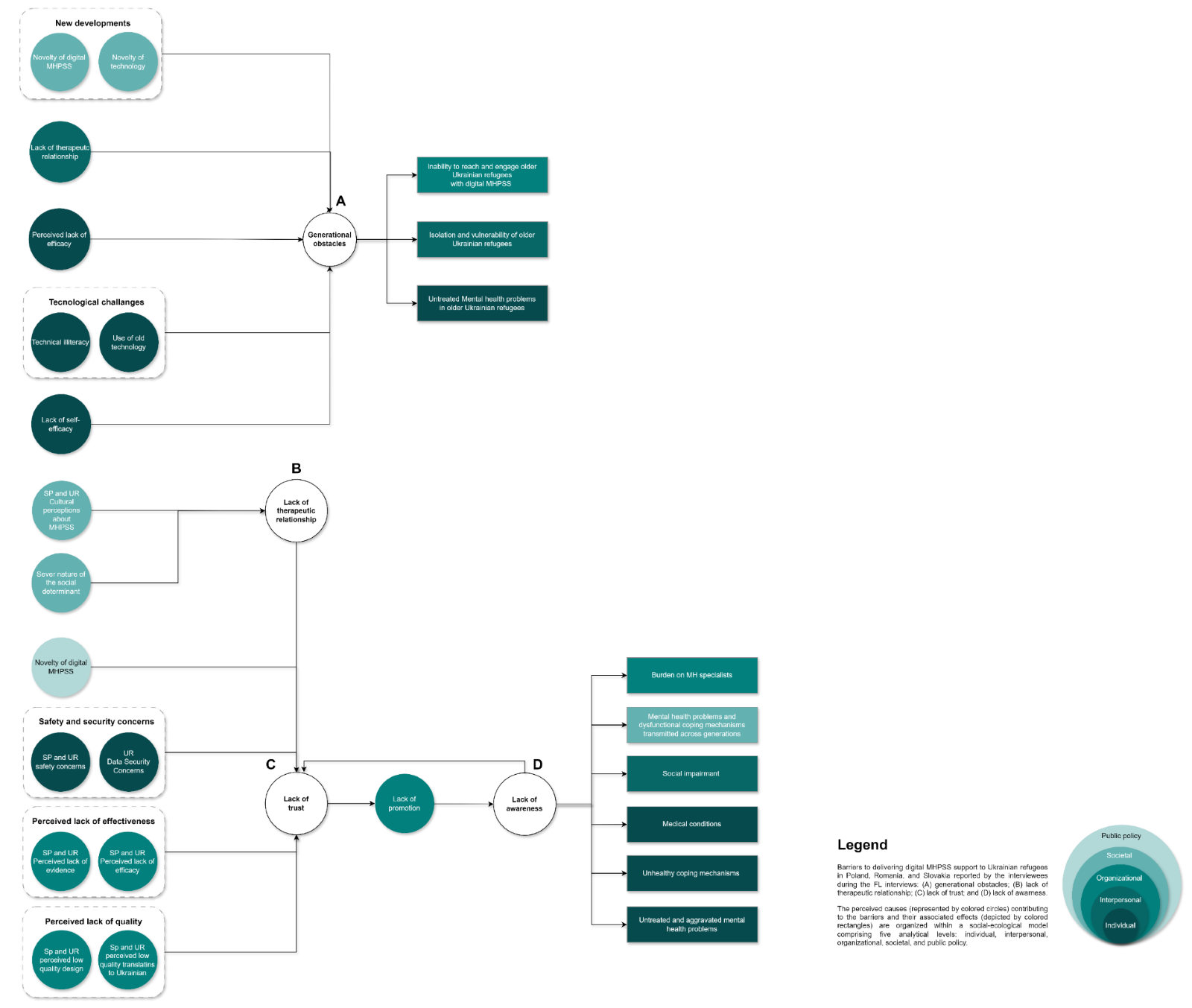


**Figure 2S.** Barriers linked to the socio-ecological framework of Bronfenbrenner.
