# Supplementary material for: Assessing local service providers’ needs for scaling up MHPSS interventions for Ukrainian refugees: Insights from Poland, Slovakia, and Romania
Source: Glob Ment Health (Camb). 2024 Dec 6;11:e119. doi: 10.1017/gmh.2024.113 (PMC11704377; doi:10.1017/gmh.2024.113)
Supplement: Peer review history

# Editor report: Recommendation: Assessing local service providers’ needs for scaling up MHPSS interventions for Ukrainian refugees: Insights from Poland, Slovakia, and Romania — R0/PR2

**DOI:** 10.1017/gmh.2024.113.pr2

**Affiliations:** Department of Global Health and Social Medicine, Harvard Medical School, United States

No accompanying comment.

# Editor report: Decision: Assessing local service providers’ needs for scaling up MHPSS interventions for Ukrainian refugees: Insights from Poland, Slovakia, and Romania — R0/PR3

**DOI:** 10.1017/gmh.2024.113.pr3

**Affiliations:** Johns Hopkins University Bloomberg School of Public Health, United States

No accompanying comment.

# Editor report: Recommendation: Assessing local service providers’ needs for scaling up MHPSS interventions for Ukrainian refugees: Insights from Poland, Slovakia, and Romania — R1/PR5

**DOI:** 10.1017/gmh.2024.113.pr5

**Affiliations:** Department of Global Health and Social Medicine, Harvard Medical School, United States

No accompanying comment.

# Editor report: Decision: Assessing local service providers’ needs for scaling up MHPSS interventions for Ukrainian refugees: Insights from Poland, Slovakia, and Romania — R1/PR6

**DOI:** 10.1017/gmh.2024.113.pr6

**Affiliations:** Johns Hopkins University Bloomberg School of Public Health, United States

No accompanying comment.
